# Supplementary material for: Conformations of a highly expressed Z19 α-zein studied with AlphaFold2 and MD simulations
Source: PLoS One. 2024 May 8;19(5):e0293786. doi: 10.1371/journal.pone.0293786 (PMC11078433; doi:10.1371/journal.pone.0293786)
Supplement: S1 File — (ZIP) [file pone.0293786.s001.zip › PLOS_ONE_SI/S16_Fig.docx]

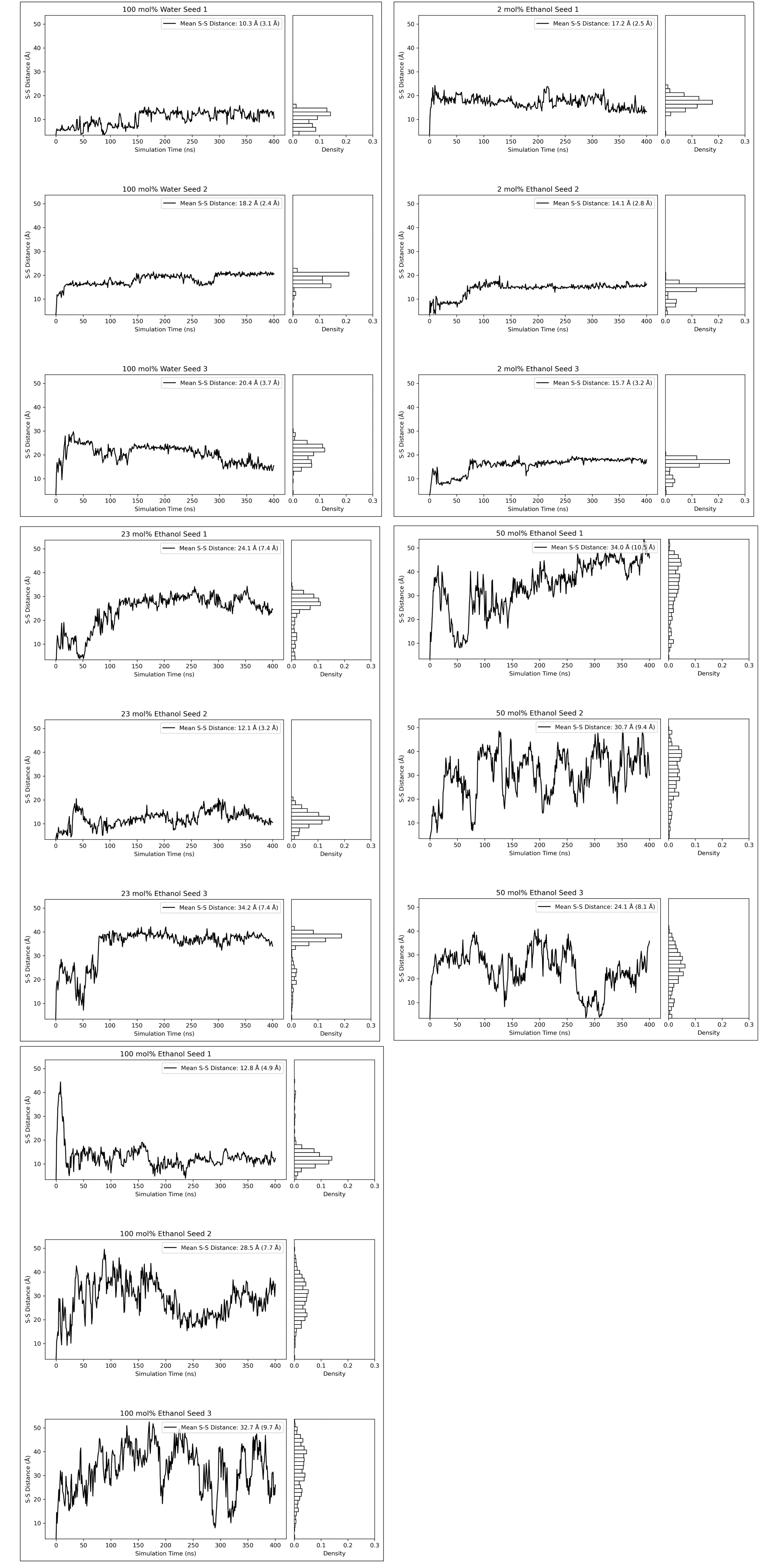


**Distance between sulfur atoms in the two cysteines across all 400 ns MD trajectories.** Mean and standard deviations in this plot are calculated using the entire 400 ns range.
