# Supplementary material for: Conformations of a highly expressed Z19 α-zein studied with AlphaFold2 and MD simulations
Source: PLoS One. 2024 May 8;19(5):e0293786. doi: 10.1371/journal.pone.0293786 (PMC11078433; doi:10.1371/journal.pone.0293786)
Supplement: S1 File — (ZIP) [file pone.0293786.s001.zip › PLOS_ONE_SI/S1_Fig.docx]

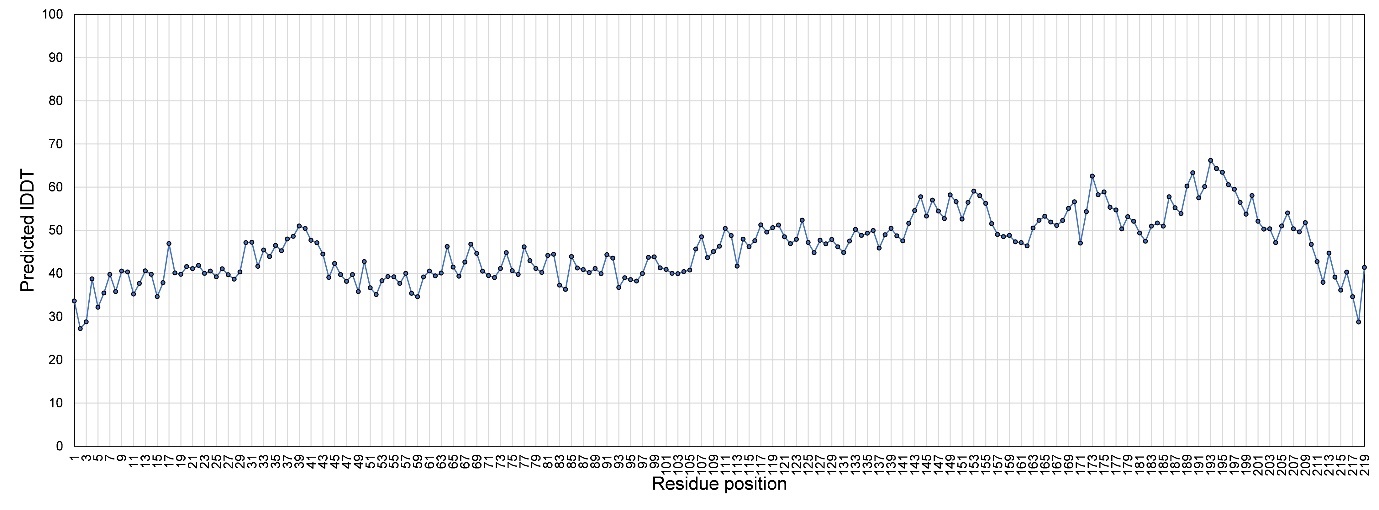


**AlphaFold2 per-residue estimate of confidence (plDDT) for the rank 1 model of α-zein cZ19C2 (UniProtKB ID: P06677).** The scale runs from 0 (minimum confidence) to 100 (maximum confidence).
