# Supplementary material for: Conformations of a highly expressed Z19 α-zein studied with AlphaFold2 and MD simulations
Source: PLoS One. 2024 May 8;19(5):e0293786. doi: 10.1371/journal.pone.0293786 (PMC11078433; doi:10.1371/journal.pone.0293786)
Supplement: S1 File — (ZIP) [file pone.0293786.s001.zip › PLOS_ONE_SI/S22_Fig.docx]

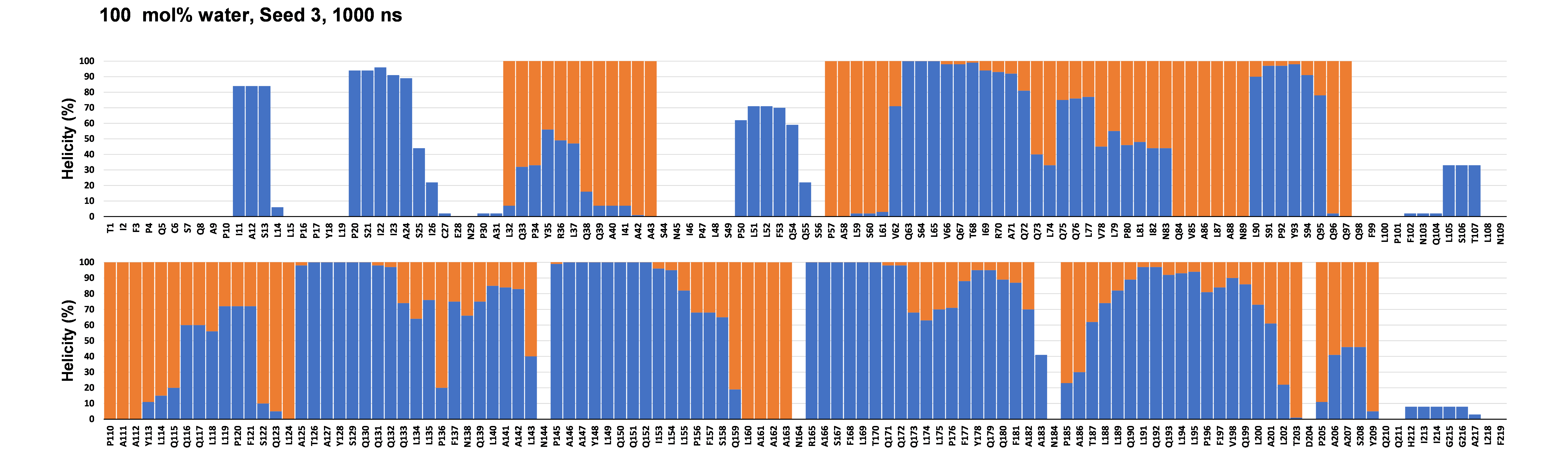


**Helicity per residue (sum of 3_10_- and α-helicity assigned by STRIDE) for the 100 mol% water 1 μs simulation.** Orange: Helicity in the initial AlphaFold2 model. Blue: Helicity during the last 100 ns.
