# Supplementary material for: Conformations of a highly expressed Z19 α-zein studied with AlphaFold2 and MD simulations
Source: PLoS One. 2024 May 8;19(5):e0293786. doi: 10.1371/journal.pone.0293786 (PMC11078433; doi:10.1371/journal.pone.0293786)
Supplement: S1 File — (ZIP) [file pone.0293786.s001.zip › PLOS_ONE_SI/S25_Fig.docx]

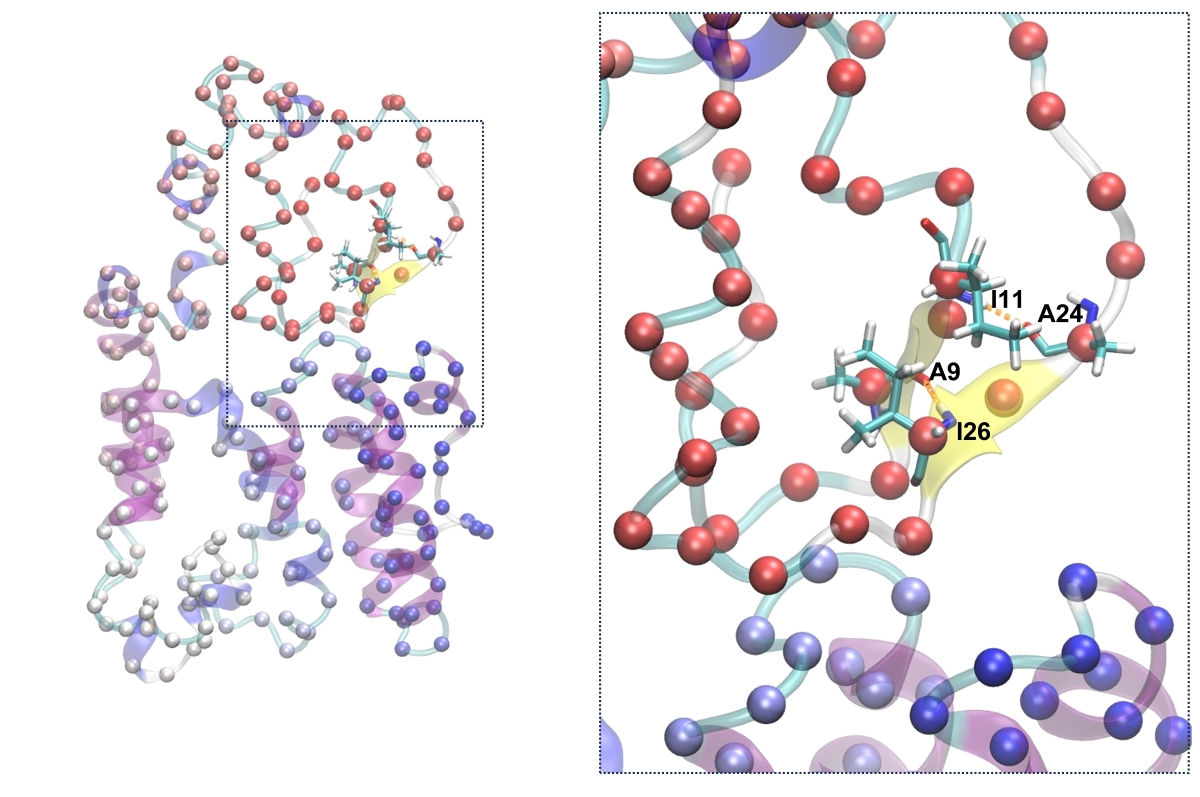


**Example of β-sheet structure in the extended 2 mol% (seed 2) ethanol simulation.** Left: MD snapshot at 1971 ns. Right: Enlarged view of the backbone hydrogen bond pairs A9/I26 and I11/A24. The protein backbone is shown in ribbon representation coloured by secondary structure. α-carbons are shown as spheres coloured by sequence position (red: N-terminal, blue: C-terminal).
