# Supplementary material for: Conformations of a highly expressed Z19 α-zein studied with AlphaFold2 and MD simulations
Source: PLoS One. 2024 May 8;19(5):e0293786. doi: 10.1371/journal.pone.0293786 (PMC11078433; doi:10.1371/journal.pone.0293786)
Supplement: S1 File — (ZIP) [file pone.0293786.s001.zip › PLOS_ONE_SI/S28_Fig.docx]

**
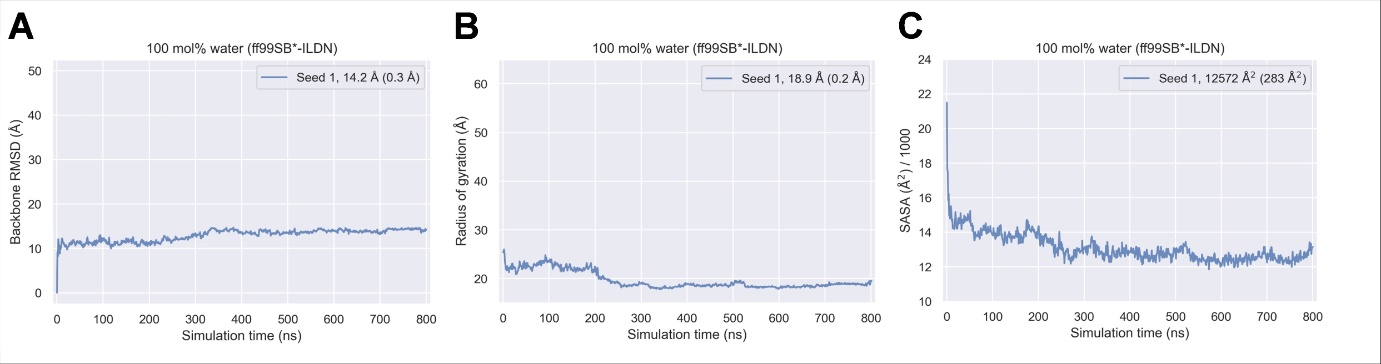
**

**MD time series for an extended GROMACS all-atom MD simulation with the ff99SB*-ILDN force field.** A: Backbone RMSD. B: Radius of gyration. C: SASA. The value given in the legend is an average over the last 100 ns, with standard deviation given in brackets.
