# Supplementary material for: Conformations of a highly expressed Z19 α-zein studied with AlphaFold2 and MD simulations
Source: PLoS One. 2024 May 8;19(5):e0293786. doi: 10.1371/journal.pone.0293786 (PMC11078433; doi:10.1371/journal.pone.0293786)
Supplement: S1 File — (ZIP) [file pone.0293786.s001.zip › PLOS_ONE_SI/S2_Fig.docx]

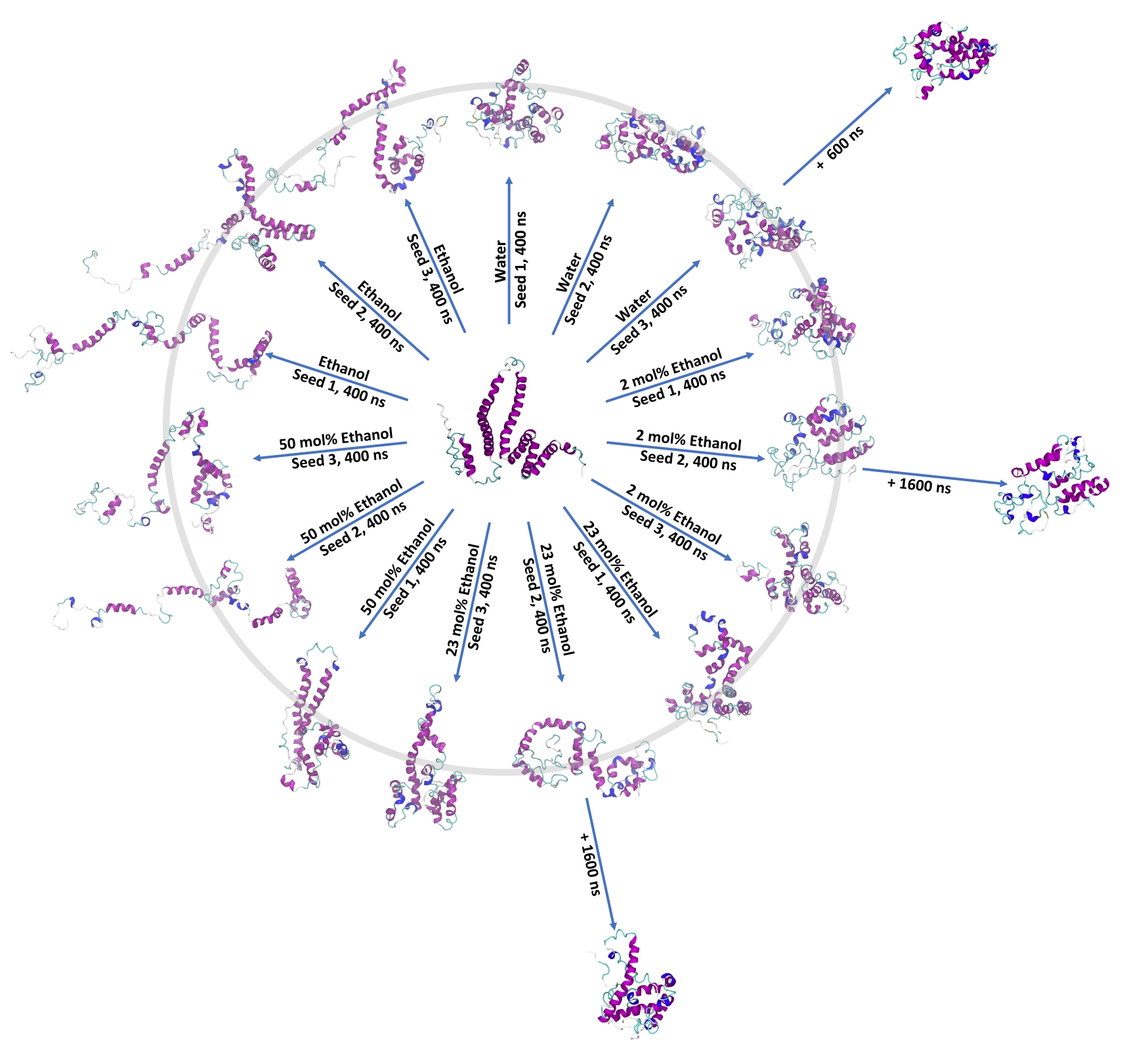


**Overview of the all-atom GROMACS simulations with the OPLS-AA/L + SPCE force fields.** 15 × 400 ns simulations of which 3 simulations were extended to at least 1 μs.
