# Supplementary material for: Conformations of a highly expressed Z19 α-zein studied with AlphaFold2 and MD simulations
Source: PLoS One. 2024 May 8;19(5):e0293786. doi: 10.1371/journal.pone.0293786 (PMC11078433; doi:10.1371/journal.pone.0293786)
Supplement: S1 File — (ZIP) [file pone.0293786.s001.zip › PLOS_ONE_SI/S36_Fig.docx]

**
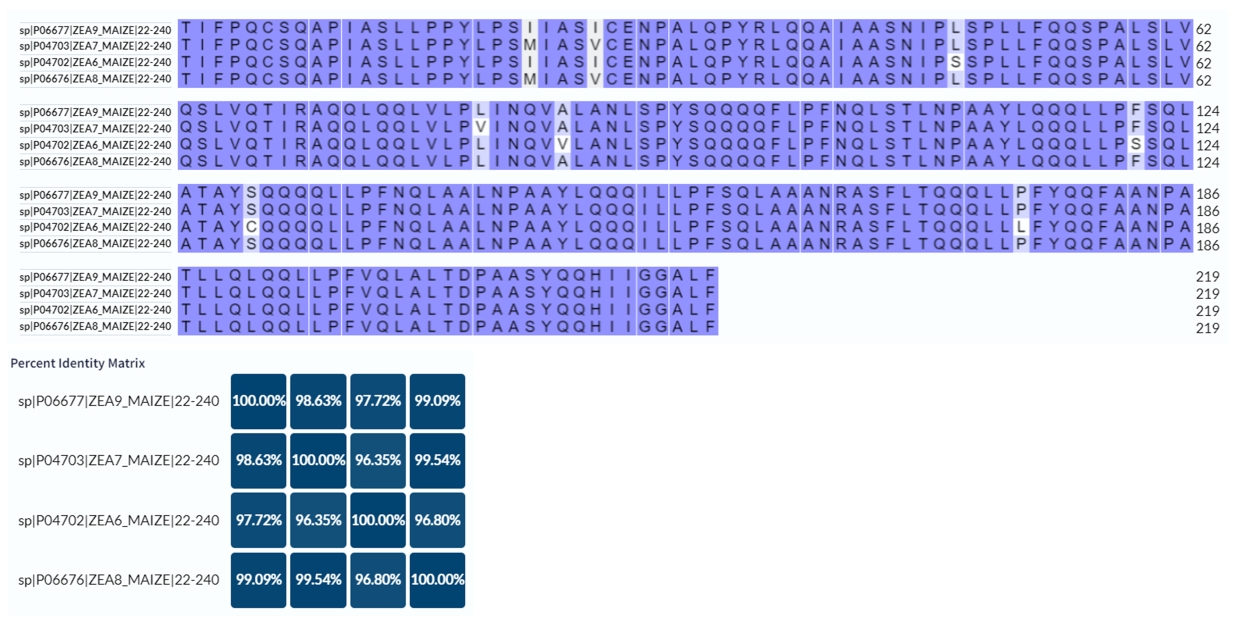
**Mature protein sequence alignment and identity matrix for P06677 (19 kDa zein 19C2), P04703 (19 kDa zein A20), P04702 (19 kDa zein M6), P06676 (19 kDa zein 19C1).
