# Supplementary material for: Conformations of a highly expressed Z19 α-zein studied with AlphaFold2 and MD simulations
Source: PLoS One. 2024 May 8;19(5):e0293786. doi: 10.1371/journal.pone.0293786 (PMC11078433; doi:10.1371/journal.pone.0293786)
Supplement: S1 File — (ZIP) [file pone.0293786.s001.zip › PLOS_ONE_SI/S4_Appendix.docx]

# **S4 Appendix. Comparison of full and partial AlphaFold2 α-zein models**

Due to the higher predicted local distance difference test (pLDDT) scores assigned to the C-terminal region of the protein, it was decided to further examine the apparent structural robustness in this region using AlphaFold2 predictions on shorter C-terminal sections of the sequence. Therefore, another set of AlphaFold2 predictions was carried out with the latest ColabFold 1.5.5 interface, which was the most recent version during the final modifications to this manuscript.

Three sequences were examined, including the mature cZ19C2 α-zein sequence, the residues from N89 to F219 (covering segments 6–12 of Figure 3A in the main text), and the segment from N144 to T203 corresponding to the C-terminal three-helix bundle.

The AF2 structures for the mature α-zein and for partial sequences are compared in Fig A, where the backbone is colored by pLDDT score. The model of the mature protein in Subfig A of Fig A generally has low pLDDT scores (mean 46, SD 6) indicating low confidence and possibly disorder. The associated predicted aligned error (PAE) matrix in Subfig D of Fig A indicates overall loose protein packing. However, as noted above, the C-terminal three-helix bundle has higher pLDDT scores. The separate AF2 models for the sequence regions (N89-F219) and (N144 -T203) are shown in Subfigs B and C, respectively. It is seen that pLDDT scores increase for smaller models. Thus, the average pLDDT for the residues N144-T203 comprising the three-helix bundle are 52 (4), 65 (4), and 71 (6), in the mature protein model, the N89-F219 model and the N144-T203 model, respectively, with standard deviations given in brackets. Despite higher pLDDT values for the partial models, their 3D structures are highly similar to the corresponding regions in the full AF2 model of the mature protein. This is evident from the comparison of the partial models to the full AF2 model as shown in Subfig G. The N89–F219 model reproduces both the long helix III (P110-L143) and the C-terminal three-helix bundle. The C-terminal three-helix bundle is also reproduced when the comprising residues N144-T203 alone are used as AF2 input. The conservation of the C-terminal α-helical bundle across full and partial models is highlighted Subfig H by superposition using the alpha-carbons of the initial residues (P145, R165, and P185) in each helix of the bundle.

In summary, AlphaFold2 demonstrates consistency in predicting the selected C-terminal sections of the cZ19C2 sequence, in particular the three-helix bundle. The structure remains highly conserved, whether modeling is based on the complete sequence or partial C-terminal sections.

**
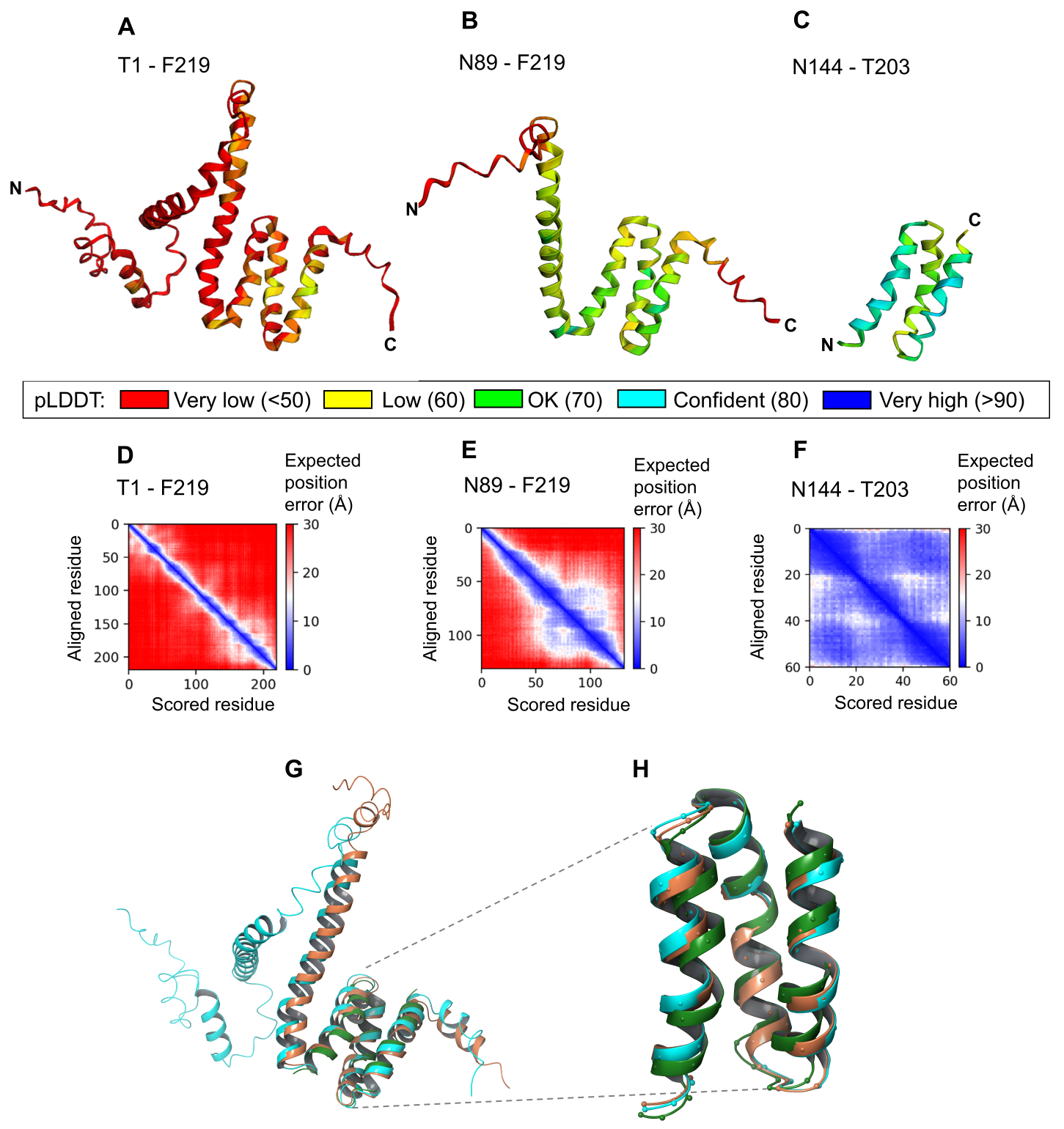
**

**Fig A. AlphaFold2 models for the mature** **cZ19C2 α-zein sequence and for progressively shorter C-terminal parts of the sequence.** A: Model for the mature protein sequence T1–F219 (UniProtKB ID P06677). B: Model for residues N89-F219. C: Model for residues N144-T203. Ribbons are colored by pLDDT score. D–F: Predicted aligned error (PAE) matrices for the models in Subfigs A-C. G: Alignment of the models in Subfigs A-C. H: Local backbone alignment of the C-terminal three-helix bundle region (residues N144-T203) of the models in Subfigs A–C. Backbone RMSD calculated relative to the three-helix bundle in the full model (cyan) were 0.6 Å for the three-helix bundle of the N89-F219 model (brown) and 1.7 Å for the N144-T203 model (green).
