# Supplementary material for: Conformations of a highly expressed Z19 α-zein studied with AlphaFold2 and MD simulations
Source: PLoS One. 2024 May 8;19(5):e0293786. doi: 10.1371/journal.pone.0293786 (PMC11078433; doi:10.1371/journal.pone.0293786)
Supplement: S1 File — (ZIP) [file pone.0293786.s001.zip › PLOS_ONE_SI/S5_Appendix.docx]

# **S5 Appendix. Early structural divergence across simulation replicates (seeds)**

To examine the structural variation around the point of divergence between MD trajectories differing by initial velocity randomization seed, MD frames were compared across replicate trajectories early in the simulations.

It was deemed most informative to compare frames from different seed trajectories at time points where the simulations had significantly propagated the initial structure, while still preserving enough similarity to the AlphaFold2 starting point to allow a meaningful graphical comparison to the latter. Time points meeting these criteria were identified using backbone RMSD calculations. Focusing on the initial 100 ns of each simulation, each subfigure of Fig A displays six RMSD plots for each system differing by ethanol concentration. The first three curves are the usual backbone RMSD plots calculated relative to the common AF2 initial structure after superposing all frames on it for each trajectory (Seed 1 - Seed 3). The three other RMSD curves were derived from these superimposed trajectories by computing the RMSD between frames at identical timepoints in distinct seed trajectories (without superimposing these frames on each other). Hence, there is a RMSD curve for each of the three possible combinations: Seed 1 vs Seed 2, Seed 1 vs Seed3, and Seed 2 vs Seed 3.

MD frame comparisons were made at the timepoint where all 6 RMSD values first simultaneously exceeded 10 Å, indicated by red vertical lines in Fig A. While this RMSD threshold was arbitrarily chosen, its magnitude ensured that the three frames compared across Seed 1, 2, and 3 differed appreciable both from the AF2 starting point and from each other. As seen, this threshold was rapidly reached in all simulations (between 8 to 16 ns).

Comparison of the structures at these time points is shown in Fig B where every subfigure corresponds to a simulation series at a specific ethanol concentration. For each seed, the initial AF2 structure is shown to the left (0 ns) and the MD frame at the specified simulation time point on the right. The orange vectors overlaid on the AF2 structure show the displacement of alpha-carbons, giving their positions in the MD snapshots on the right. For clarity, the vectors are only shown for the alpha-carbons displaying the largest displacements between the two structures, identified as those exceeding the average alpha-carbon displacement by one standard deviation. The average alpha-carbon displacements and standard deviations are listed in Table A.

Fig B shows that the general structure of the AF2 model remains discernible in the early MD frames, yet there is notable divergence across different seeds. Residues at the N-terminal, in particular, frequently show significant displacement. This agrees with the loose packing in this region and the relative lack of well-defined secondary structure for the initial N-terminal residues. Residues near the turn between Helix II and Helix III (see main text Fig 3) are also substantially displaced in several frames, reflecting the instability of the long helices and the flexible residues in the connecting turn. The absence of a consistent preferred direction for structural deformation is evident in the changing direction of displacement vectors for similar regions across seeds. Regions that are rarely highlighted by displacement arrows include the C-terminal three-helix bundle, noted in the main text for its relatively high stability in most simulations.

Thus again, the overall loose packing of the AF2 structure, particularly in the mentioned regions, renders the AF2 starting point susceptible to random perturbations in the simulations. These perturbations propagate throughout the MD simulations contributing to the distinct endpoints noted in the main text.

**
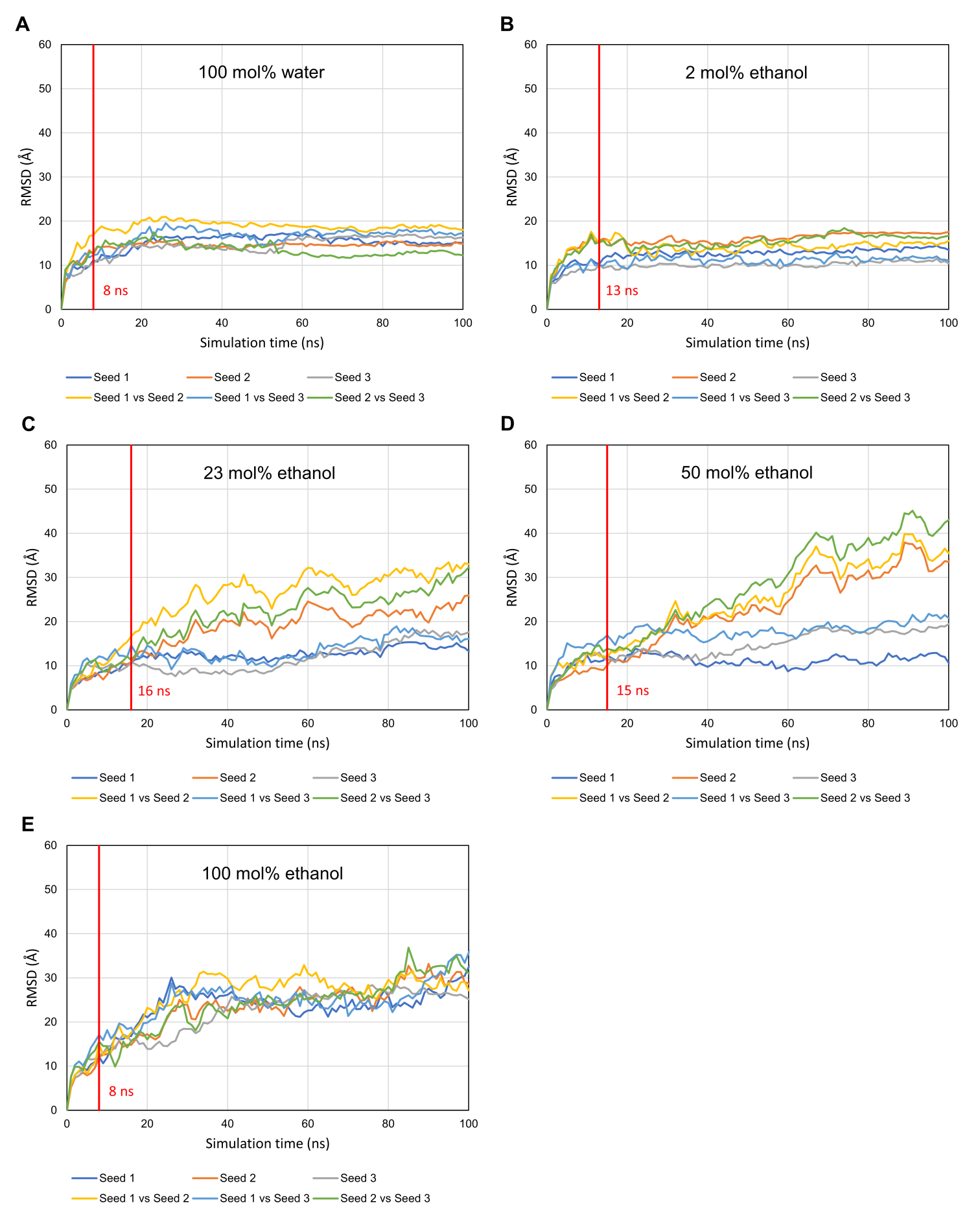
**

**Fig A. Backbone RMSD curves for the first 100 ns of each simulation.** In each plot, the first three curves are conventional backbone RMSD plots, calculated with reference to the shared AF2 initial structure, with all frames superimposed individually on the starting structure for each trajectory (Seed 1 - Seed 3). The other three RMSD curves were generated from these superimposed trajectories by determining the RMSD between frames at corresponding timepoints in different seed trajectories, without aligning these frames with each other. The red vertical line in each plot indicates the first timepoint at which all six RMSD curves exceed 10 Å.

**Table A. Average alpha-carbon displacements between initial AF2 structure and superimposed MD frames.** Units are Å. Standard deviations are given in brackets.

|  | **100 mol% water** | **2 mol% EtOH** | **23 mol% EtOH** | **50 mol% EtOH** | **100 mol% EtOH** |
| --- | --- | --- | --- | --- | --- |
| **Time (ns)** | 8 | 13 | 016 | 15 | 8 |
| **Seed 1** | 10.1 (4.7) | 9.4 (4.3) | 9.6 (5.1) | 10.0 (7.3) | 10.7 (5.8) |
| **Seed 2** | 11.3 (5.3 | 14.2 (6.6) | 9.7 (4.7) | 8.9 (4.5) | 10.1 (6.7) |
| **Seed 3** | 9.8 (4.1) | 8.7 (5.4) | 10.0 (4.2) | 10.5 (4.1) | 10.9 (6.2) |


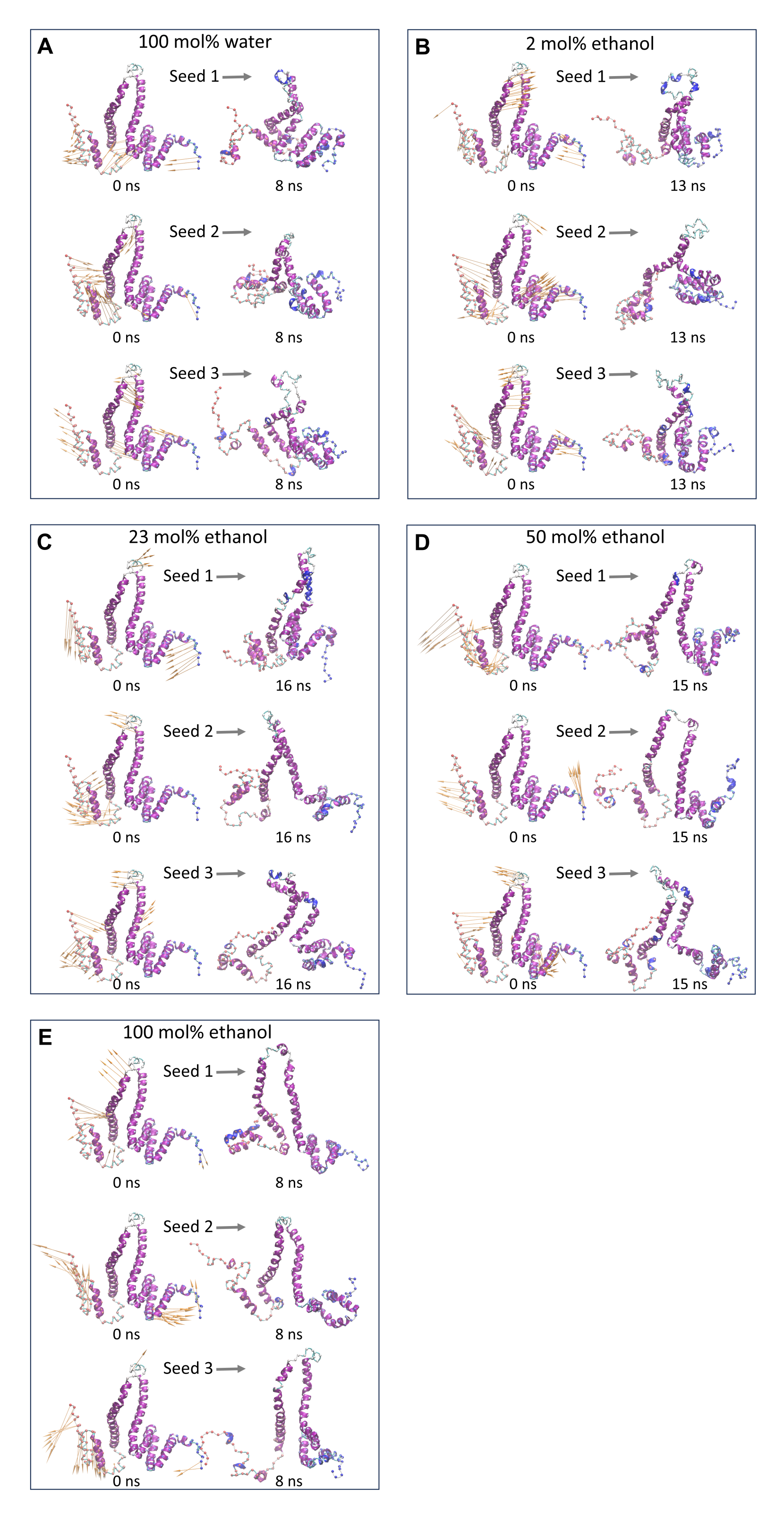


**Fig B.** **Early structural divergence of simulations differing by seed.** For each seed at each condition (ethanol concentration) the initial AF2 structure is shown on the left and the MD frame at the designated point of divergence on the right. Ribbons are coloured by secondary structure. Alpha-carbons are shown as spheres coloured by residue position (red: N-terminal, Blue: C-terminal). Orange vectors mark alpha-carbon displacements between the two structures that exceed the average of all alpha-carbon displacements between the two structures by one standard deviation (see Table A).
