# Supplementary material for: Conformations of a highly expressed Z19 α-zein studied with AlphaFold2 and MD simulations
Source: PLoS One. 2024 May 8;19(5):e0293786. doi: 10.1371/journal.pone.0293786 (PMC11078433; doi:10.1371/journal.pone.0293786)
Supplement: S1 File — (ZIP) [file pone.0293786.s001.zip › PLOS_ONE_SI/S6_Appendix.docx]

# **S6 Appendix. Simulations on the isolated C-terminal region N144-F219**

The isolated C-terminal region of the AlphaFold2 model consisting of residues N144 to F219 was studied separately with a 1 μs MD simulation in water using Desmond. This region encompassed the C-terminal three-helix bundle (N144-T203), the short helix VII (P205-Y209), and the disordered residues (Q210-F219)­. Prior to simulation, the termini were neutralized by N-terminal acetylation and C-terminal amidation, aiming to minimize the impact of electrostatic interactions with the termini in this smaller model system. The force field parameters and settings were identical to those for other Desmond simulations presented in the main text, see the Methods section for details.

The time development of spatial structure and secondary structure is illustrated in Fig A. The top panel (A) shows MD frames of the protein sampled at 100 ns intervals, with the backbone colored by secondary structure. Gln and Asn residues are highlighted by sticks in green and orange, respectively for the alpha- and beta-carbons. Panel B shows the secondary structure per residue. Time series for backbone RMSD, radius of gyration, and solvent accessible surface area are shown in Fig B. RMSD for both the C-terminal three-helix bundle alone and the full system are shown in blue and orange, respectively.

It is evident from the structural snapshots and the RMSD curves that the initial AF2 structure of the C-terminal helical bundle is generally well-conserved throughout the 1 μs simulation, although some rearrangements occur. The secondary structure plot (Fig A, panel B) reveals a tendency for reduction of α-helicity at the C-termini of the individual helices making up the bundle, with the last 3-4 residues in each helix typically becoming less α-helical toward the end of the simulation. For instance, residues L200-T203 in the last helix (Helix VI) of the bundle adopt coil or turn structure. Likewise, turn structure is induced in the C-terminal residues L161-L163 of the first helix (Helix IV) in the bundle, as well as in F181-A182 of the second helix (Helix V). The helicity of the short C-terminal fragment (Q210-F219) is dynamic and fluctuates among residues over the course of the simulation, and showing variations in type (α-helix vs. 3-10 helix).

During MD, the model as a whole adopts a more compact packing, as reflected by the significant drop in both radius of gyration and SASA around 100 ns (see panels B and C of Fig B). The structural implications are evident from Fig C, where comparisons of the initial structure and a representative MD snapshot near the end of the simulation at 999.4 ns are presented in panels A and B. During the simulation, Helix IV and Helix VI of the bundle are driven together, as particularly clear from the top view in panel A of Fig C. The increased proximity of the helices can also be noticed in the side view in Panel B of Fig C. The overall compaction furthermore involves the arrangement of the C-terminal residues P205-Q210 in a transverse orientation, packed against the collective surface formed by the associated helices IV and VI. This can clearly be seen in Panel A of Fig C. The compaction is associated with the establishment of a hydrogen bond network involving the side chains of Q192 and Q193 in Helix VI, Q210 in Helix VII, and Q152 in Helix IV. This hydrogen bonding network, occurring between different helices, potentially contributes to stabilizing their association, and is depicted in more detail in Panel C of Fig C.


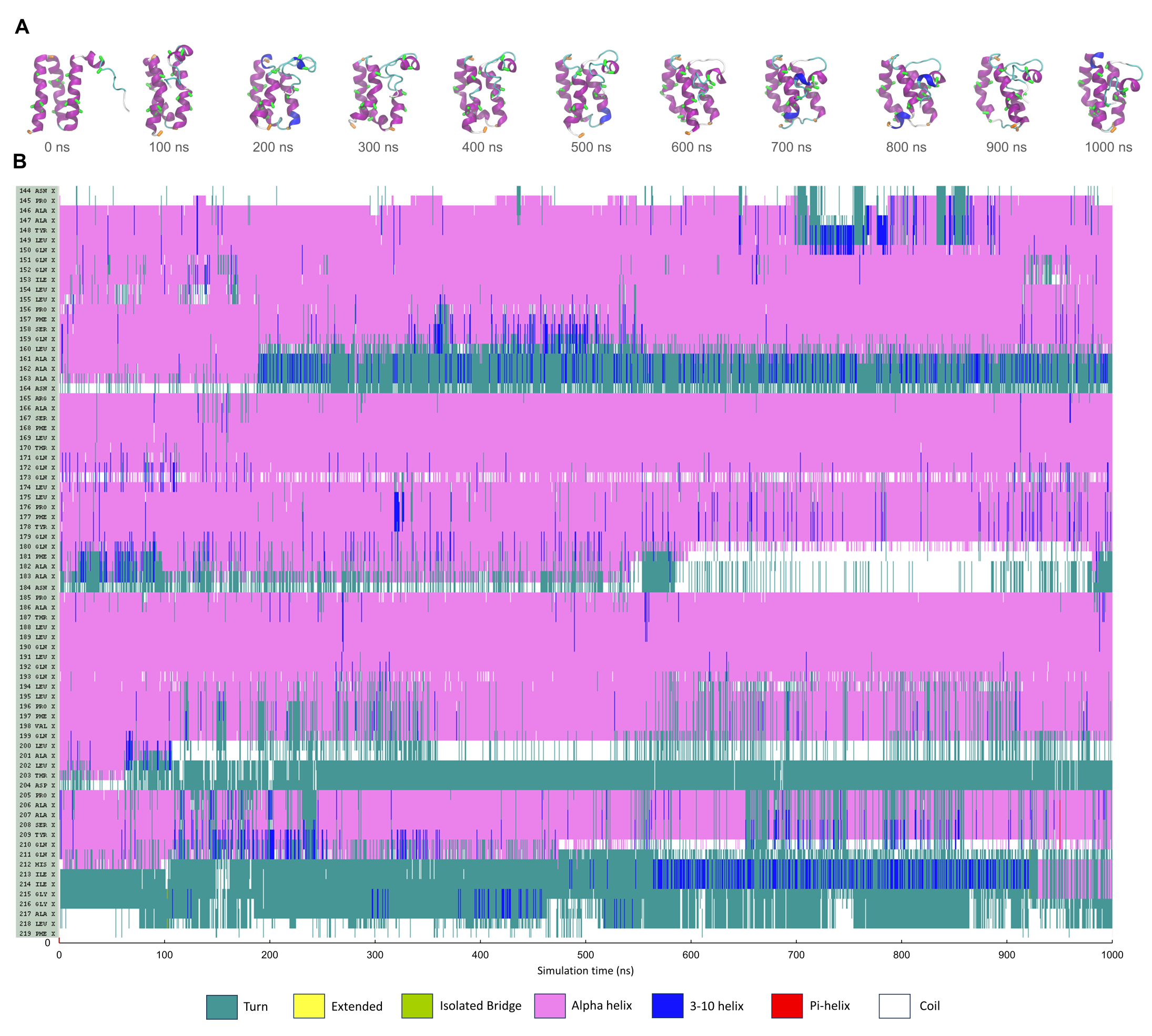


**Fig A.** **Structural snapshots and secondary structure for MD simulation of the N144-F219 model.** Panel A: MD frames sampled at 100 ns intervals. Gln and Asn positions are highlighted by sticks in green and orange for the alpha- and beta-carbons. Backbone is colored by secondary structure. Panel B: Secondary structure per residue versus simulation time. The legend below explains the VMD-style secondary structure colors used.


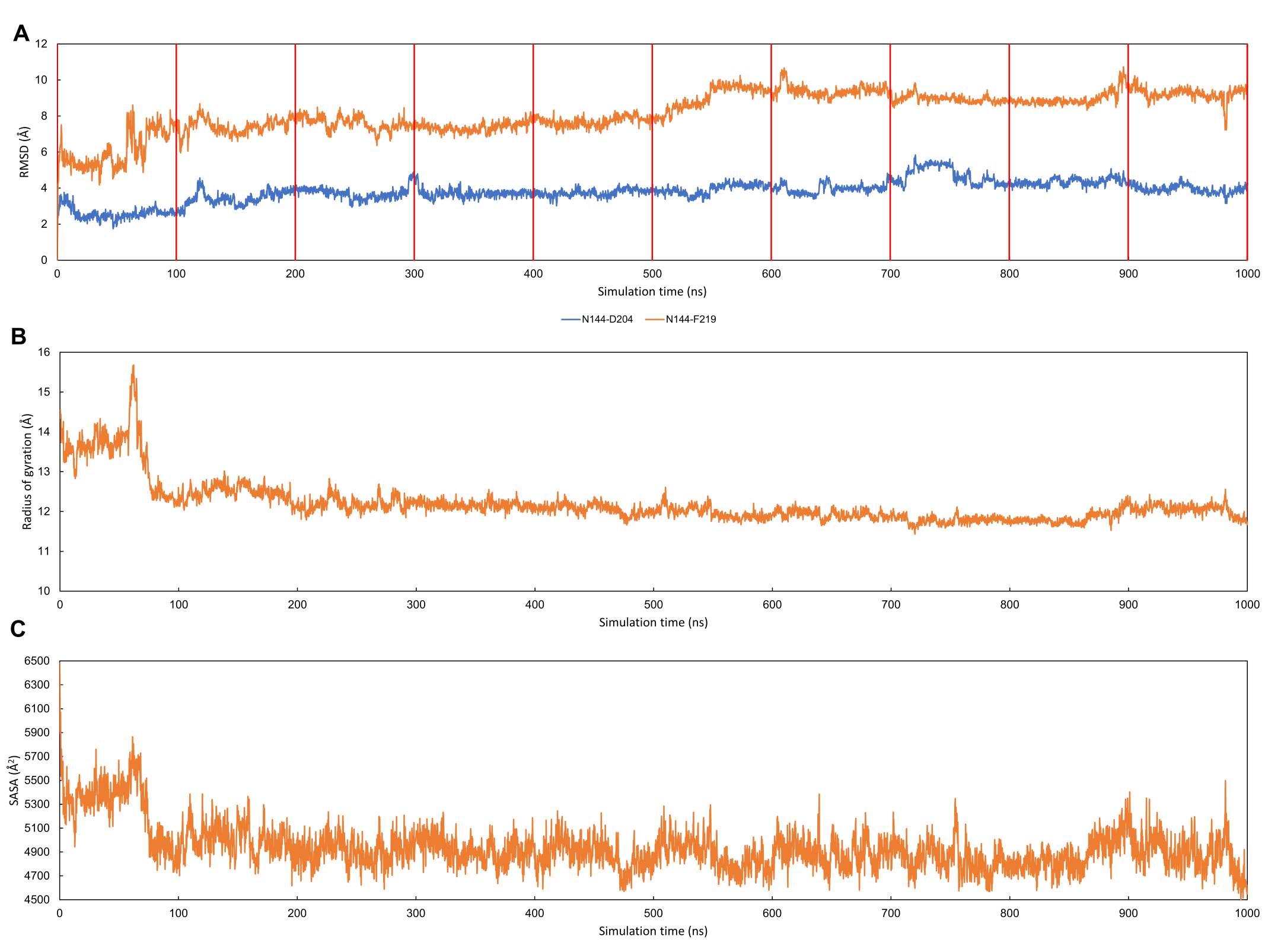


**Fig B.** **RMSD, radius of gyration, and SASA time series for MD simulation of the N144-F219 model.** Panel A: Backbone RMSD for the N144-D204 segment (blue) and for the whole N144-F219 model (orange). Red vertical lines indicate time points at which the structures in panel A of Fig A were obtained. Panel B: Radius of gyration. Panel C: Solvent accessible surface area (SASA).

**
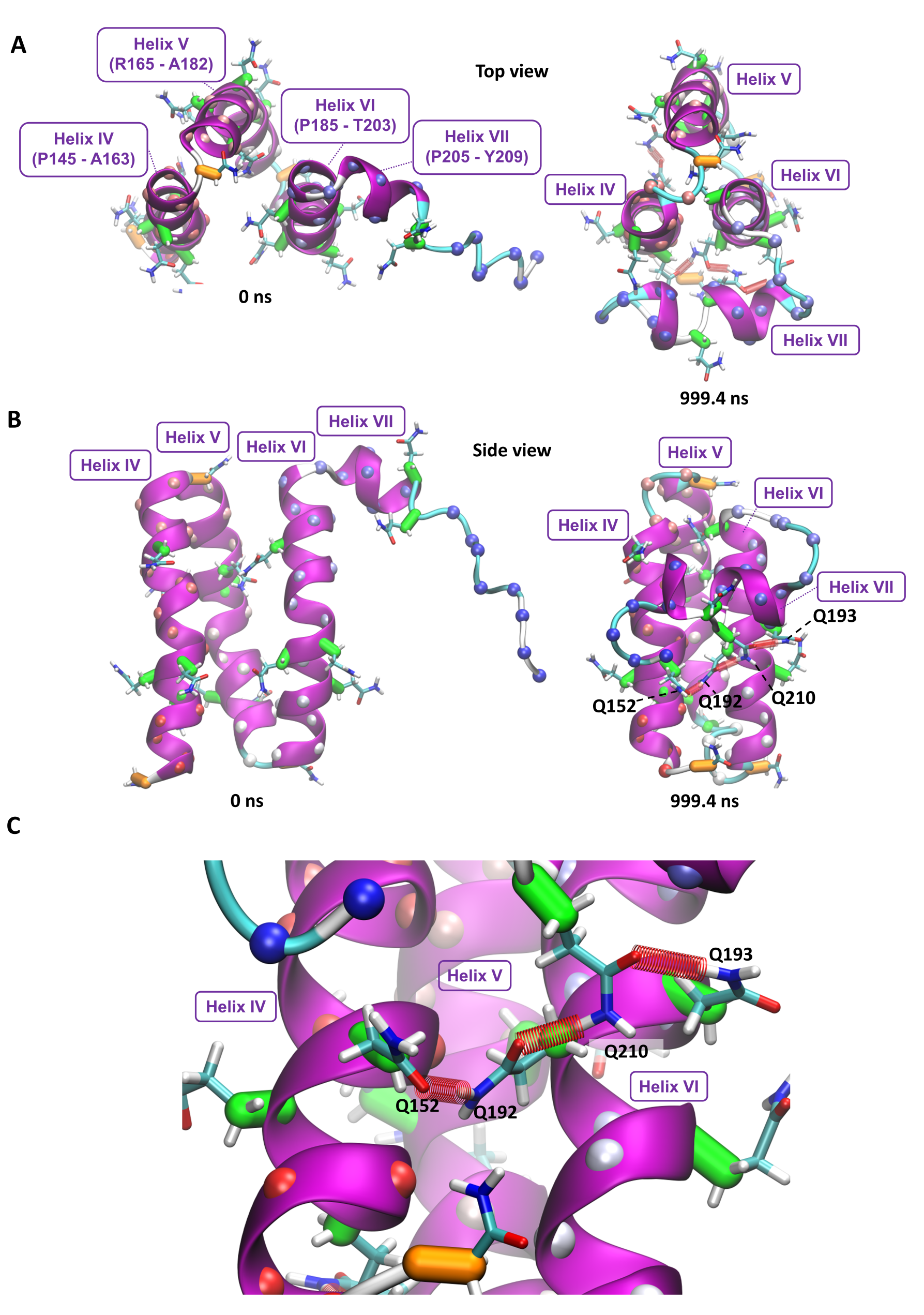
**

**Fig C.** **Structure of the N144-F219 model before and after MD simulation.** Left: Initial structure. Right: Structure for MD frame 4999 (999.4 μs). The backbone is colored by secondary structure. Alpha-carbons are shown as spheres, colored by residues position. Gln (green) and Asn (orange) residues are shown in stick representation, with the alpha- and beta-carbons highlighted in a thicker cylinder representation. Panel A and B show top views and side views, respectively, of the structures. Panel C shows a magnified view of the network of glutamine side-chain-side-chain hydrogen bonds, highlighted by red springs.
