# Supplementary material for: Conformations of a highly expressed Z19 α-zein studied with AlphaFold2 and MD simulations
Source: PLoS One. 2024 May 8;19(5):e0293786. doi: 10.1371/journal.pone.0293786 (PMC11078433; doi:10.1371/journal.pone.0293786)
Supplement: S1 File — (ZIP) [file pone.0293786.s001.zip › PLOS_ONE_SI/S6_Fig.docx]

**
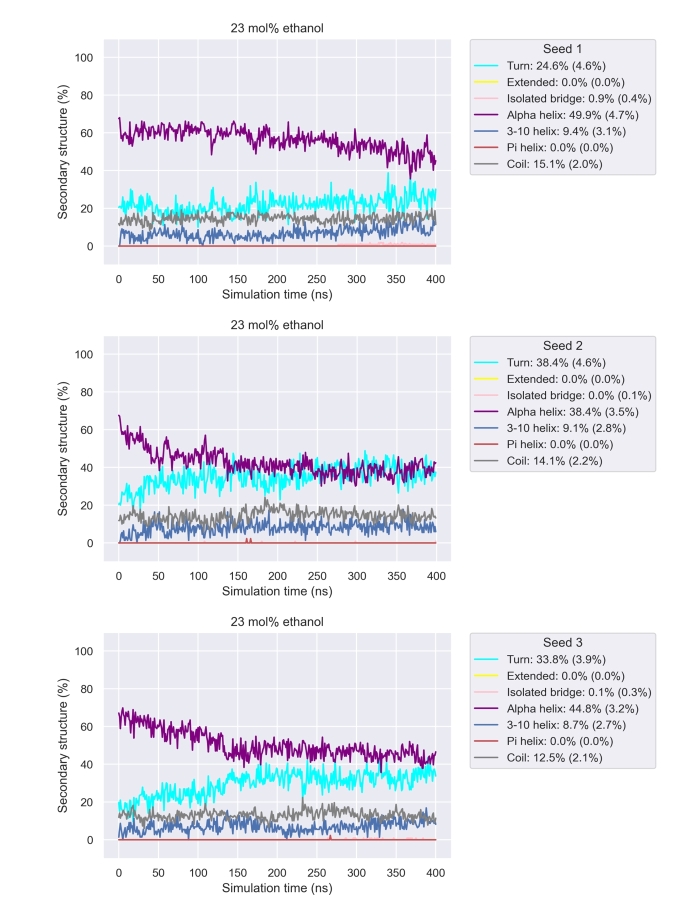
**

**Time series for secondary structure for 23 mol% ethanol 400 ns MD simulations.** Values given in the legend for each seed are averages over the last 100 ns of each simulation, with standard deviations given in brackets.
