# Supplementary figures and images for: Conformations of a highly expressed Z19 α-zein studied with AlphaFold2 and MD simulations
Source: PLoS One. 2024 May 8;19(5):e0293786. doi: 10.1371/journal.pone.0293786 (PMC11078433; doi:10.1371/journal.pone.0293786)

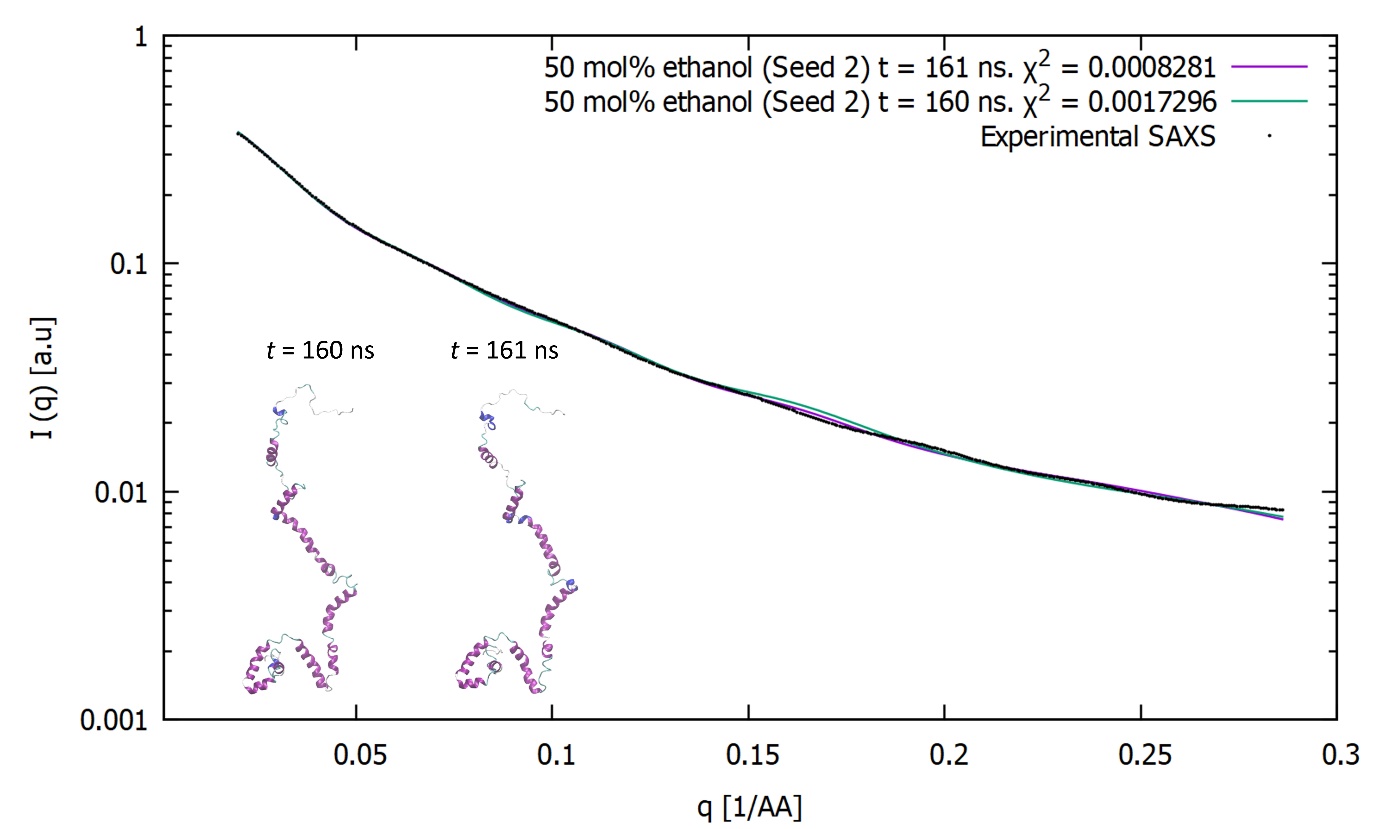


**SAXS predictions.** Predictions for 50 mol% ethanol showing the two best fits at *t* = 161 ns and *t* = 160 ns.

Supplement: S1 File — (ZIP) [file pone.0293786.s001.zip › PLOS_ONE_SI/S18_Fig.docx]

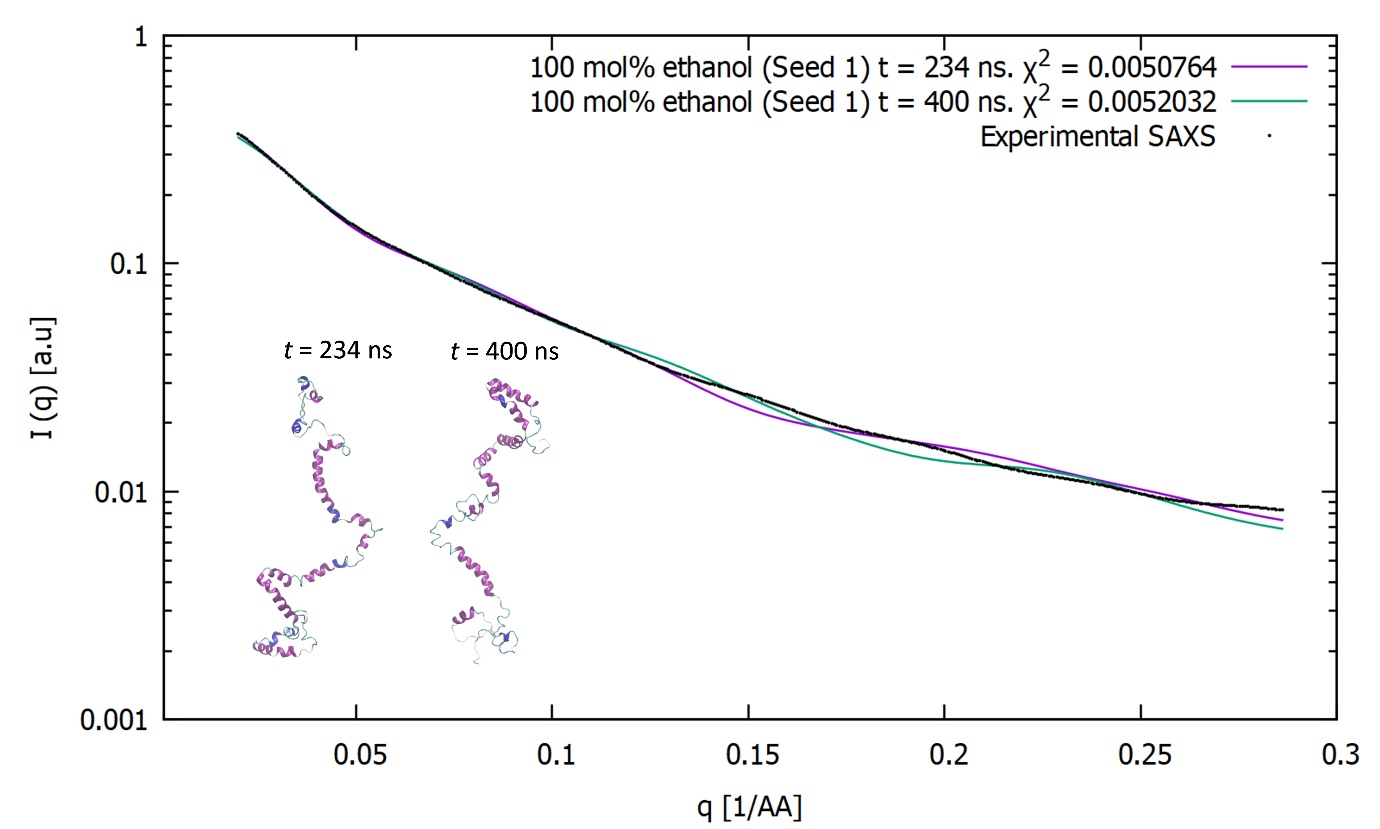


**SAXS predictions.** Predictions for 100 mol% ethanol showing the two best fits at *t* = 234 ns and *t* = 400 ns.

Supplement: S1 File — (ZIP) [file pone.0293786.s001.zip › PLOS_ONE_SI/S19_Fig.docx]

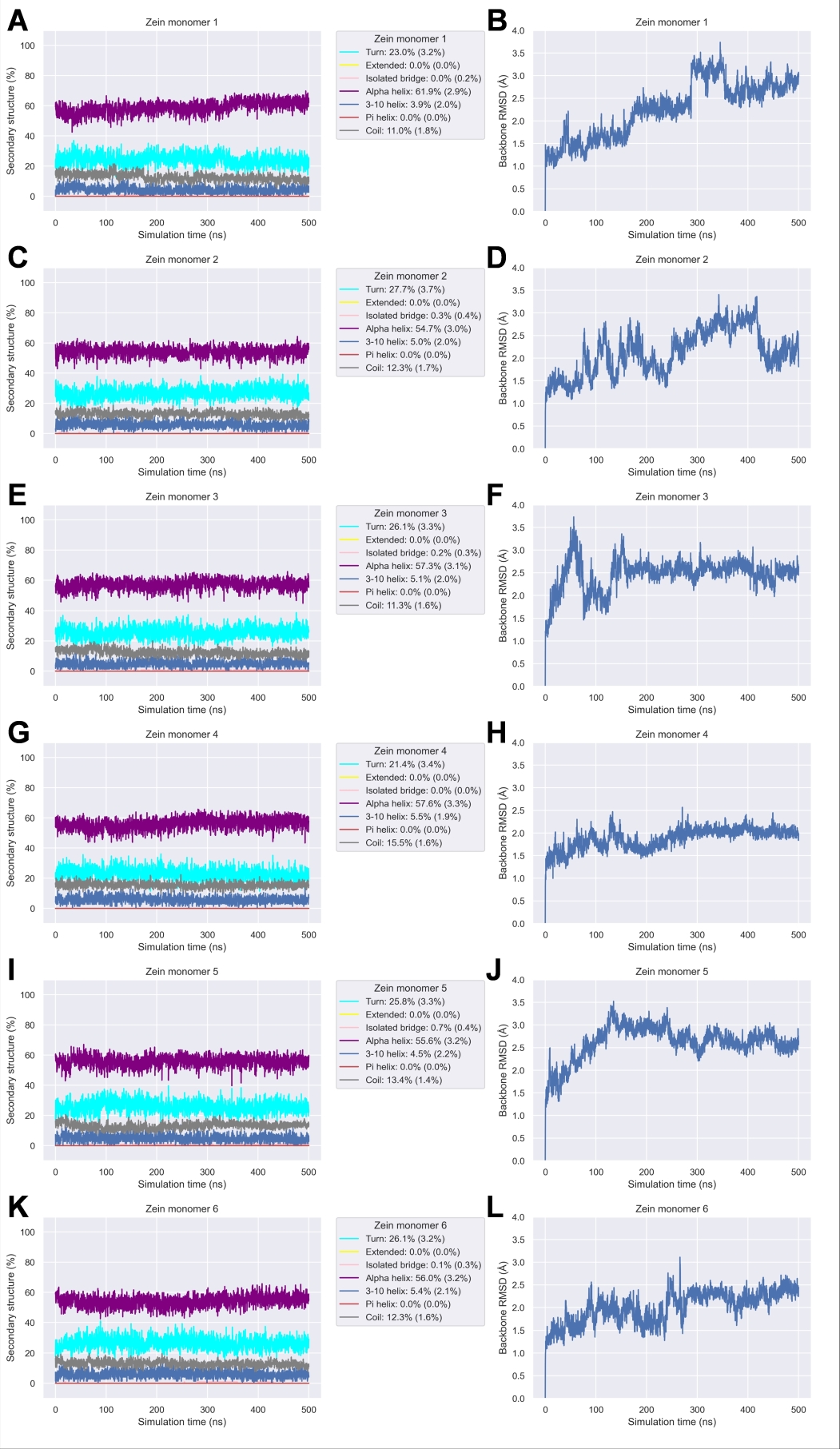


**Secondary structure and backbone RMSD for all-atom MD simulations of 6 zein copies in a water box.**

Supplement: S1 File — (ZIP) [file pone.0293786.s001.zip › PLOS_ONE_SI/S35_Fig.docx]

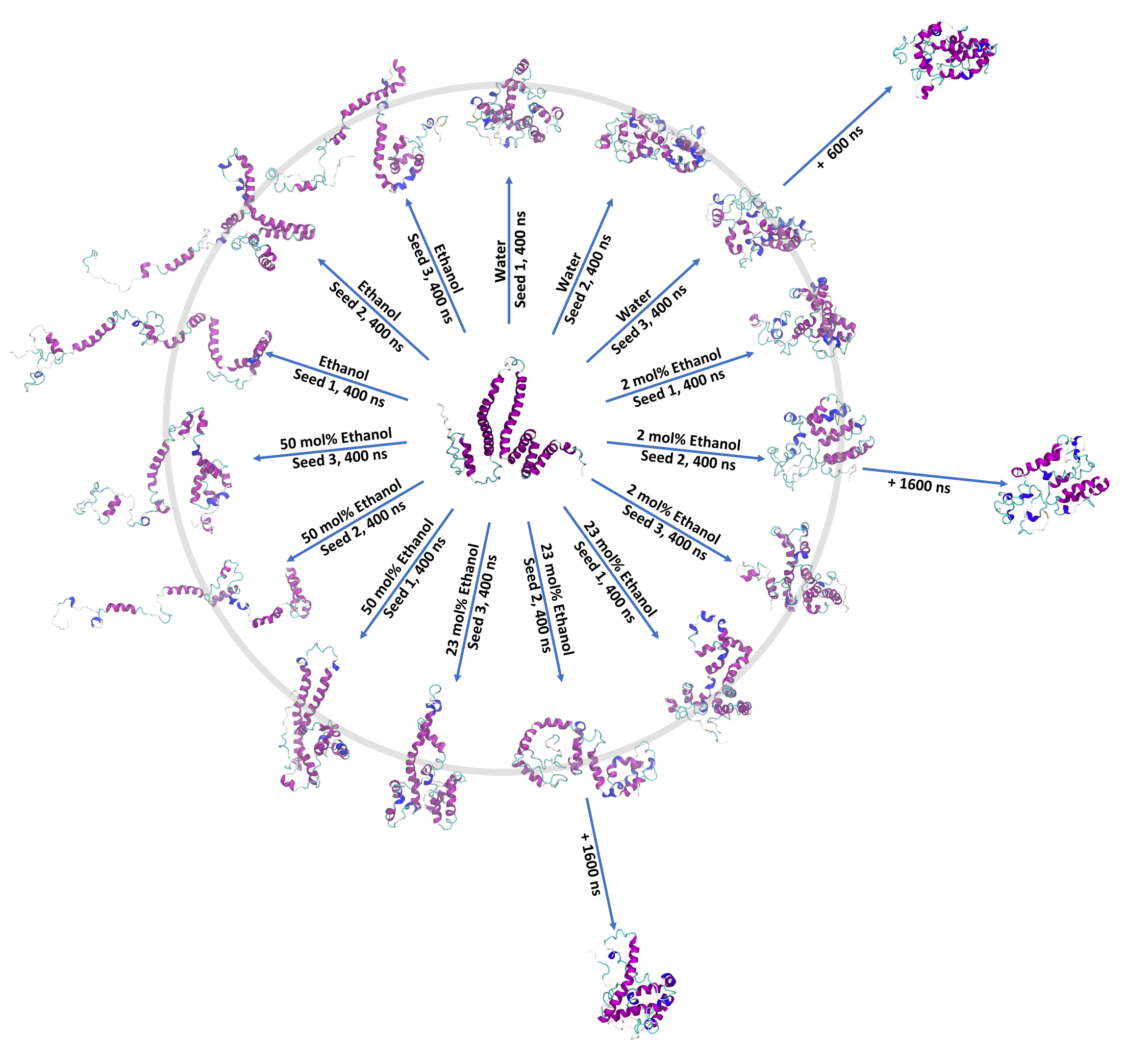

Supplement: S1 Striking image — (TIF) [file pone.0293786.s002.tif]
