# Supplementary material for: Conformations of a highly expressed Z19 α-zein studied with AlphaFold2 and MD simulations
Source: PLoS One. 2024 May 8;19(5):e0293786. doi: 10.1371/journal.pone.0293786 (PMC11078433; doi:10.1371/journal.pone.0293786)

1) Geragthy D et al (1981) a-zein repeat consensus

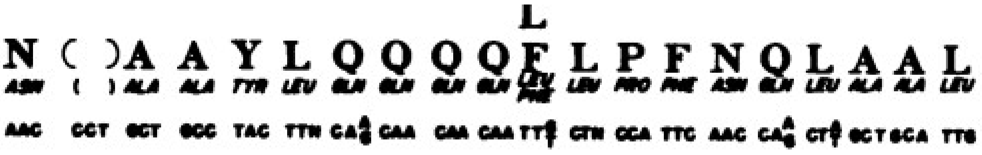

2) Argos P. et al (1982) a-zein repeat consensus

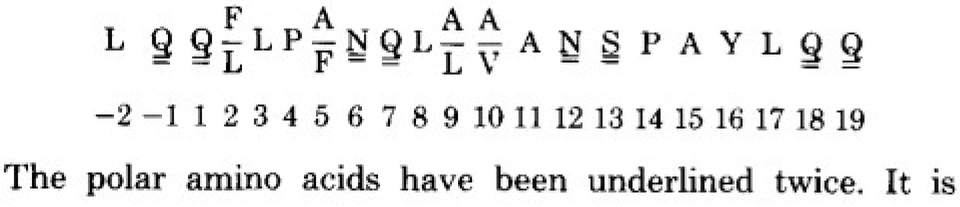

3) AlphaFold2 full-length a-zein 3D model with per-residue quality LDDT scores

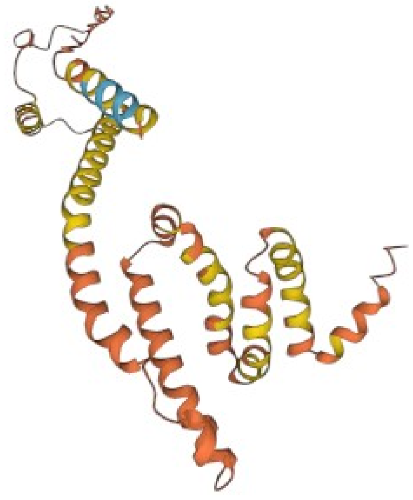

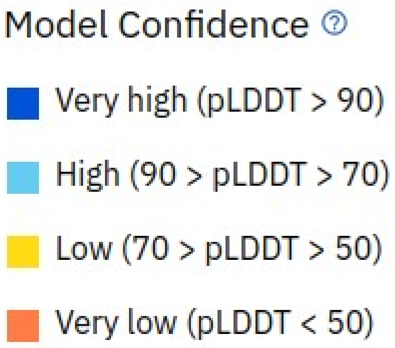

(continue)

3) AlphaFold2 full-length a-zein 3D model PAE matrix

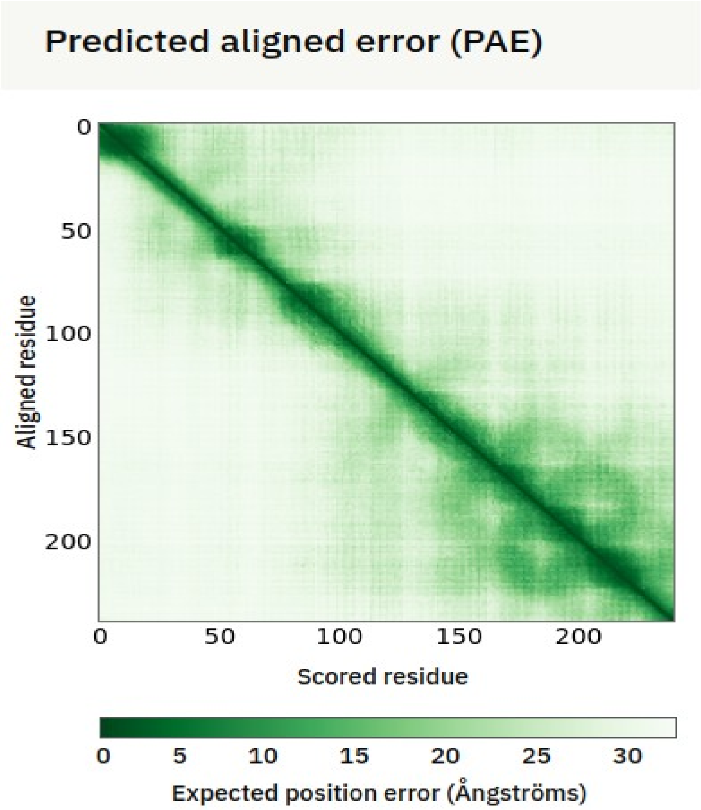

Supplement: Attachment — Submitted filename: reviewing_figure_attachments.pdf [file pone.0293786.s003.pdf]
