# Supplementary material for: Conformations of a highly expressed Z19 α-zein studied with AlphaFold2 and MD simulations
Source: PLoS One. 2024 May 8;19(5):e0293786. doi: 10.1371/journal.pone.0293786 (PMC11078433; doi:10.1371/journal.pone.0293786)
Supplement: Attachment — Submitted filename: Response_to_Reviewers.docx [file pone.0293786.s004.docx]

**Response to reviewers (PONE-D-23-34203)**

Dear Dr. Christensen,

Thank you for submitting your manuscript to PLOS ONE. After careful consideration, we feel that it has merit but does not fully meet PLOS ONE’s publication criteria as it currently stands. Therefore, we invite you to submit a revised version of the manuscript that addresses the points raised during the review process.

==============================

As you can see below, Reviewers #1, #2 and #4 are entirely or partially positive on your work. Although Reviewer #4 recommended major revisions, the amount of work required is not dramatic and would critically improve the quality of this Manuscript. However, Reviewer #3 strongly recommended major revisions (points 1.1, 1.2 and 1.3) and in particular what brings the most perplexity and should be principally addressed is point 1.2) Second major revision: quality of the a-zein AF2 predicted 3D model. Considering the tremendous amount of work that has been conducted, my suggestion is to do analyses of the C-ter three a-helix bundle only in addition to those already obtained on the full-length and discuss further the results in light of the general points raised out by this Reviewer on the model quality.

This manuscript is of interest for this Journal and the scientific community, as Reviewer #2 pointed out, it is a comprehensive study describing the landscape of *possible* structures for further investigation.
==============================

Please submit your revised manuscript by Feb 16 2024 11:59PM. If you will need more time than this to complete your revisions, please reply to this message or contact the journal office at [plosone@plos.org](mailto:plosone@plos.org). When you're ready to submit your revision, log on to <https://www.editorialmanager.com/pone/> and select the 'Submissions Needing Revision' folder to locate your manuscript file.

Please include the following items when submitting your revised manuscript:

- A rebuttal letter that responds to each point raised by the academic editor and reviewer(s). You should upload this letter as a separate file labeled 'Response to Reviewers'.
- A marked-up copy of your manuscript that highlights changes made to the original version. You should upload this as a separate file labeled 'Revised Manuscript with Track Changes'.
- An unmarked version of your revised paper without tracked changes. You should upload this as a separate file labeled 'Manuscript'.

If you would like to make changes to your financial disclosure, please include your updated statement in your cover letter. Guidelines for resubmitting your figure files are available below the reviewer comments at the end of this letter.

If applicable, we recommend that you deposit your laboratory protocols in protocols.io to enhance the reproducibility of your results. Protocols.io assigns your protocol its own identifier (DOI) so that it can be cited independently in the future. For instructions see: [https://journals.plos.org/plosone/s/submission-guidelines#loc-laboratory-protocols](https://eur02.safelinks.protection.outlook.com/?url=https%3A%2F%2Fjournals.plos.org%2Fplosone%2Fs%2Fsubmission-guidelines%23loc-laboratory-protocols&data=05%7C02%7Cnjc%40chem.ku.dk%7Cc02134b5f56b41ee7aa608dc0be62eea%7Ca3927f91cda14696af898c9f1ceffa91%7C0%7C0%7C638398330752024687%7CUnknown%7CTWFpbGZsb3d8eyJWIjoiMC4wLjAwMDAiLCJQIjoiV2luMzIiLCJBTiI6Ik1haWwiLCJXVCI6Mn0%3D%7C3000%7C%7C%7C&sdata=hD%2FhqQM1WHBJjI4NvlrD5u65fGsZAfTdyBO9pekbWXE%3D&reserved=0). Additionally, PLOS ONE offers an option for publishing peer-reviewed Lab Protocol articles, which describe protocols hosted on protocols.io. Read more information on sharing protocols at [https://plos.org/protocols?utm_medium=editorial-email&utm_source=authorletters&utm_campaign=protocols](https://eur02.safelinks.protection.outlook.com/?url=https%3A%2F%2Fplos.org%2Fprotocols%3Futm_medium%3Deditorial-email%26utm_source%3Dauthorletters%26utm_campaign%3Dprotocols&data=05%7C02%7Cnjc%40chem.ku.dk%7Cc02134b5f56b41ee7aa608dc0be62eea%7Ca3927f91cda14696af898c9f1ceffa91%7C0%7C0%7C638398330752024687%7CUnknown%7CTWFpbGZsb3d8eyJWIjoiMC4wLjAwMDAiLCJQIjoiV2luMzIiLCJBTiI6Ik1haWwiLCJXVCI6Mn0%3D%7C3000%7C%7C%7C&sdata=m0wLJEdKbd%2Fs%2BKL4oqCfFeOKfVsKbbLT1SaECbjAR4w%3D&reserved=0).

We look forward to receiving your revised manuscript.

Kind regards,

Matteo De March
Academic Editor
PLOS ONE

Journal Requirements:

When submitting your revision, we need you to address these additional requirements.

1. Please ensure that your manuscript meets PLOS ONE's style requirements, including those for file naming. The PLOS ONE style templates can be found at 
<https://journals.plos.org/plosone/s/file?id=wjVg/PLOSOne_formatting_sample_main_body.pdf> and 
<https://journals.plos.org/plosone/s/file?id=ba62/PLOSOne_formatting_sample_title_authors_affiliations.pdf>

2. Please note that PLOS ONE has specific guidelines on code sharing for submissions in which author-generated code underpins the findings in the manuscript. In these cases, all author-generated code must be made available without restrictions upon publication of the work. Please review our guidelines at <https://journals.plos.org/plosone/s/materials-and-software-sharing#loc-sharing-code> and ensure that your code is shared in a way that follows best practice and facilitates reproducibility and reuse.

5. Review Comments to the Author

***Reviewer #1****: In this work, the Author characterized the structural ensemble of alpha-zein cZ19C2 using a model predicted by AlphaFold2 (AF2), followed by all-atom and coarse-grained MD simulations on the model. Started with a loosely packed initial AF2 model, the protein exhibited high conformational heterogeneity in simulations, suggesting that ensemble-based approaches are preferred over single native-structure based ones. Valuable observations were obtained – including expanded conformations in high ethanol conditions.

Overall, this manuscript presented substantial and valuable results in many aspects. There are, however, several points that require attention:*

**Author:** I thank the reviewer for feedback on the manuscript.

***Reviewer****: 1. The MD simulations suggest the C-terminal helical bundle has a relatively stable structure compared to the rest of the protein, Figures 14A and B may indicate a disordered N-terminal region and a more stable C-terminal core region. Also, sequence from segment 6 to C-terminal end agrees well with the consensus repeat (Figure 3).
In light of these, I wonder if AF2 prediction on the shortened sequence (either from segment 6 to C-terminus or helices 4-6) will yield a structure with better confidence. If yes, it may be interesting to model these regions separately before their integration, perhaps in future studies.*

**Author:** I agree with the reviewer that it is interesting to investigate AF2 predictions on partial C-terminal sequences, i.e., from segment 6 to the C-terminus and helices 4 - 6. Hence, I have carried out additional AF2 predictions for both of these partial sequences. The results are presented and discussed in a new appendix for the revised manuscript, named: S4 Appendix. Comparison of full and partial AlphaFold2 α-zein models. To summarize, when each of these regions was modeled independently, higher local model confidence estimates (pLDDT) were observed. However, upon superimposing the models, the structure of the C-terminal α-helical bundle remained highly similar across all three models. This indicates robustness, or internal consistency, in the AF2 predictions.

The following text has been added to the revised manuscript, Line 409:

“To further examine the apparently more structurally well-defined C-terminal part of the protein, the full-sequence AlphaFold2 model was compared with partial-sequence AlphaFold2 models built from N89-F219 (segments 6 to 12 in Fig 3A) and N144-T203 (the C-terminal three-helix bundle). As shown in S4 Appendix, structural superposition revealed the α-helical bundle to be highly similar across the three models, indicating internal consistency in the AlphaFold2 predictions.”

***Reviewer****: 2. What is the rationale of using OPLS forcefield? Does it provide a more accurate modelling on proteins with disordered regions such as the one studied here?*

**Author:** The main rationale was to use a forcefield that strikes a balance between generality and accuracy in representing protein structures and dynamics. OPLS-AA/L emerged as a suitable choice. While OPLS-AA/L is not specifically tailored for disordered regions, its long history of use to study diverse biomolecular systems made it a pragmatic choice for investigating proteins with varying structural characteristics (such as this AlphaFold2 model). Reference simulations at 100 mol% water and 24 mol% ethanol concentrations were studied with the newer field ff99SB*-ILDN, to serve as a supplementary evaluation of certain trends observed in the OPLS-AA/L simulation series.

***Reviewer****: 3. It may be interesting to follow and elucidate conformational changes and key molecular interactions near the point of divergence in different replicates at early time points in the simulation. Besides Figure 14, a detailed comparison at early time points between replicates, in various conditions, will enhance the manuscript.*

**Author:** I agree that inspection of early structural divergence between different simulation replicates may be interesting. To address this, I have included a new appendix with the revision examining this aspect, titled “S5 Appendix. Early structural divergence across simulation replicates (seeds)”. In summary the analysis shows that structural divergence is substantial already early in the simulations (before 20 ns) consistent with the loose packing of the AlphaFold2 model. The most loosely packed regions of the model tend to undergo the largest displacements, with no obvious preference for the direction of structural deformation. These findings are expected and in line with the results previously discussed in the main text. The following has been added to the main text of the revised manuscript at Line 523:

“The divergence between trajectories differing by seed was substantial already early in the simulations, see S5 Appendix.”

***Reviewer****: Minor points concerned with general layout and illustrations:

4. In my opinion, the Abstract contains a lot of technical quantitative descriptions, that would be better replaced with more concise descriptions on major findings and their implications, in order to attract readers.*

**Author:** In response to the reviewer's suggestions, the abstract has been modified in the revised manuscript. The updated abstract is presented below:

”α-zeins are amphiphilic maize seed storage proteins with material properties suitable for a multitude of applications e.g., in renewable plastics, foods, therapeutics and additive manufacturing (3D-printing). To exploit their full potential, molecular-level insights are essential. The difficulties in experimental atomic-resolution characterization of α-zeins have resulted in a diversity of published molecular models. However, deep-learning α-zein models are largely unexplored. Therefore, this work studies an AlphaFold2 (AF2) model of a highly expressed α-zein using molecular dynamics (MD) simulations. The sequence of the α-zein cZ19C2 gave a loosely packed AF2 model with 7 α-helical segments connected by turns/loops. Compact tertiary structure was limited to a C-terminal bundle of three α-helices, each showing notable agreement with a published consensus sequence. Aiming to chart possible α-zein conformations in practically relevant solvents, rather than the native solid-state, the AF2 model was subjected to MD simulations in water/ethanol mixtures with varying ethanol concentrations. Despite giving structurally diverse endpoints, the simulations showed several patterns: In water and low ethanol concentrations, the model rapidly formed compact globular structures, largely preserving the C-terminal bundle. At ≥ 50 mol% ethanol, extended conformations prevailed, consistent with previous SAXS studies. Tertiary structure was partially stabilized in water and low ethanol concentrations, but was disrupted in ≥ 50 mol% ethanol. Aggregated results indicated minor increases in helicity with ethanol concentration. β-sheet content was consistently low (~1%) across all conditions. Beyond structural dynamics, the rapid formation of branched α-zein aggregates in aqueous environments was highlighted. Furthermore, aqueous simulations revealed favorable interactions between the protein and the crosslinking agent glycidyl methacrylate (GMA). The proximity of GMA epoxide carbons and side chain hydroxyl oxygens simultaneously suggested accessible reactive sites in compact α-zein conformations and pre-reaction geometries for methacrylation. The findings may assist in expanding the applications of these technologically significant proteins, e.g., by guiding chemical modifications.”

***Reviewer****: 5. There are many brief sections in Methods that should be merged. For example: “GROMACS All-atom Simulations” & “GROMACS All-atom Simulations Protocol”.*

**Author:** In response to this point, I have removed headings for the following brief sections in Methods: “All-atom Simulations with the OPLS-AA/L Force Field”, “GROMACS all-atom simulation protocol”, and “All-atom Simulations with the ff99SB*-ILDN Force Field”.

***Reviewer****: 6. Typological error at line 318 “…the sequence bas been…”*

**Author:** I have corrected this error in the revised manuscript.

***Reviewer****: 7. Similar to 5, the Result part of the manuscript would benefit from merging related sections, if appropriate. In general, the readability could be better improved with a more condensed layout and descriptive section titles.*

**Author:** I have merged related parts of the Results section when deemed appropriate in the revision. For example, the following headings for brief sections have been removed: “Sequence”, “Initial Scan for Self-assembly Domains”, “AlphaFold2 Model”. The content has been merged in the new section titled “α-zein Sequence and Initial Structural Model” with minor adjustments to the relevant text. Furthermore, I have introduced an additional heading level in order to reorganize several subsections, e.g., under “400 ns All-atom MD Simulations”, aiming to provide a more condensed layout. All changes can be tracked in the revised manuscript.

***Reviewer****: 8. In my opinion, some main figures could be combined to reduce redundancy, for example Figures 4-8. There are also too many Supplementary Figures (36), this number should be reduced*

**Author:** I acknowledge the reviewer’s opinion about reducing the number of figures, but I would like to note that my use of figures aligns with the journal's liberal figure policy i.e.:

“There are no restrictions on word count, number of figures, or amount of supporting information.” (https://journals.plos.org/plosone/s/submission-guidelines).

The number of figures can only be reduced by (a) excluding figures or (b) by merging multiple figures. I have already considered both approaches while preparing the manuscript. For instance, the reviewer suggests merging Figures 4 – 8, which already consist of subfigures. Further merging of Figures 4 – 8 would lead to a very large figure, even after excluding certain elements that could be considered redundant. The resolution of such a figure would be lower relative to the current individual figures, since the journals “Figure File Requirements” states a maximum figure file size of 10 MB. In my opinion, sacrificing figure resolution for space is not a satisfactory tradeoff here. Similarly, omitting figures solely to condense the manuscript seems to contradict the relatively free-format ethos of the journal. Given these considerations, I have decided not to reduce the number of figures.

***Reviewer****: 9. In Figure 11, the uniform orange “bars” can be confusing, they may be better represented by other means.*

**Author:** I agree that there is (always) room for improving graphical representations. However, the current one represents the best effort on my part, and I believe it provides a serviceable depiction of helicity versus sequence. The reviewer has not offered a specific explanation for finding the orange bars potentially confusing. This leads me to speculate that the figure's description could be the culprit. Therefore, I have revised the Fig 11 caption using the alternative formulation below to improve clarity:

“The height of the blue bars in the foreground indicates the helicity in percent from MD simulations (sum of 3-10 and α-helicity of the last 100 ns of each 400 ns simulation averaged over all 15 × 400 ns MD simulations.) The uniform orange bars in the background indicate positions of α-helical residues in the AlphaFold2 model.”

***Reviewer****: 10. To help visual comparison of these highly heterogenous structures in most figures, coloring the ribbon presentation of protein structures by residue numbering (such as S2 Appendix C) may further enhance the quality of these illustrations.*

**Author:** I appreciate and share the reviewer’s focus on molecular visualization. Depiction of 3D protein structures in 2D is always challenging and further complicated by differing opinions on the most effective mode of representation. However, I wish to emphasize that I have already tried to optimize the current figures, driven by my decision to accentuate specific aspects of certain figures as they are discussed. For instance, in Figures 4 to 8, the idea is to convey the relative tightness of MD ensembles and their overall shapes, while simultaneously giving an impression of secondary structure. Hence the ribbons are colored by secondary structure rather than residue numbering. These particular figures are not intended for detailed differentiation among individual conformers. However, other figures were indeed made to present molecular structure in more detail. In those cases, I share the reviewer’s sentiment regarding the usefulness of coloring by residue position. The reviewer may perhaps have overlooked that I have used residue position coloring of C-alpha atoms in Figures 3, 12, 14, and 15. While more subtle than coloring the entire ribbon by secondary structure, the figures in their current resolution do in fact allow simultaneously conveying residue position (by C-alpha color) and secondary structure (by ribbon color). However, if the resolution were to be reduced, e.g., via the aforementioned figure merging to decrease the overall figure count, it may compromise the simultaneous depiction of both properties

I have become aware, thanks to the feedback from this reviewer and another, that I have not stated the use of secondary structure color coding in several figure captions. Consequently, I have added the following text to the relevant figures:

“The ribbon representation is colored by secondary structure (see legend in Fig 3B).”

Also, importantly, the first submission lacked this legend for secondary structure coloring. This has been included in the revised Fig 3B.

***Reviewer #2****: This paper leverages Alphafold2 to generate a model of a representative alpha-zein and then performs molecular dynamics simulations in modelling structures in different concentrations of solvent and makes some attempt to identify interfaces that may exist in oligomers. It correlates results of the simulations with SAXS data and reports good correlations for some endpoints. The length of the simulations is adequate to converge on structures within runs. However the endpoints are diverse. It is not clear whether the structures are reasonable or relevant to structures adopted in a physiologic setting, but the author has not claimed that this is the case. As such, it is a comprehensive study describing the landscape of *possible* structures for further investigation. Given that alpha-zeins may be useful biomaterials this information may inform production pathways. The manuscript is clearly written, the data supports the modest claims, and comparisons of the current study with past literature are outlined.*

**Author:** The reviewer is thanked for feedback on the manuscript. The positive remarks on clarity, support for claims made, and recognition of literature comparisons are valued. I acknowledge the observation regarding simulation endpoint diversity. As stated, I did explicitly not claim relevance to physiological conditions, and I appreciate the reviewer’s recognition of this important nuance. The reviewer’s recognition of the work as a comprehensive study of possible structures for further investigation fully aligns with the aim of the study.

***Reviewer #3****: 2023/12/15

Conformations of a highly expressed Z19 a-zein studied with AlphaFold2 and MD simulations

Niels Joan Christensen
University of Denmark


The main goal of the manuscript is to study the conformation landscape of the cZ19C2 a-zein, i.e. a 240 aa protein which is expressed in maize. The protein shows various applications as a biopolymer. Bioinformatics sequence analysis and molecular modeling are both valuable approach to study the a-zein since the protein has been refractory to 3D structure resolution by classical experimental methods (NMR, X-ray crystallography).*


***Reviewer:*** *General remarks:

The english of the manuscript is of good quality. The a-zein is a particularly challenging protein to study since no homologous structure is available in the protein data bank (PDB). The author did a tremendous amount of work, especially molecular dynamics (MD) simulations.
When writing a scientific article, concision is appreciated. The author presents 19 figures in the main manuscript and 36 in Supplementary information (SI). For the sake of clarity, 7 or 8 figures in the main and 15 in SI should not be exceeded otherwise the manuscript may appear bushy. The graphical quality of the structural pictures were rather poor and improvements are needed. Residues of Figure 3a were also difficult to read, for instance I had difficulties to distinguish Ile (I) and Leu (L) residues. Did the author make screen captures of pictures ? If yes, it should be avoided for publication because the output is of low graphical quality.
I was interested by the article because a-zeins are challenging bioinformatics targets and I found original that some MD simulations were carried out in ethanol rather than water as it is traditional. I was surprised that the author wrote a long conclusion whereas the manuscript has no discussion. This must be corrected. I understood that the author’s main claim was that the protein adopts a packed conformation in water and an elongated one in ethanol. However I have both questions and major concerns about the data which is presented and I wish the author will find them valuable to increase the quality of the manuscript.*

**Author:** I thank the reviewer for the substantial feedback on the manuscript. I acknowledge the reviewer's view on the manuscript style and inquiries about the data, and I will provide a detailed response to them in the point-by-point response below. Two critical points deserve attention already here:

First, a significant portion of the reviewer's critique seems to arise from a misinterpretation of the study's aim: It seems there is an assumption that the objective is to propose and study a model of α-zein in a native-like form, representative of its biological environment (i.e., in the solid state inside protein bodies). Consequently, the reviewer provides extensive comments on perceived challenges with the study, focusing on the perceived shortcomings of the AF2 model due to its pLDDT and PAE metrics. However, the manuscript clearly states that the primary aim is not to model a physiological state of α-zein. Instead, the focus is on computational characterization of various solution states of α-zein that might have technological or practical relevance. Only on a more speculative note might some of the MD conformations warrant future investigation for their potential resemblance to compact α-zein states in biological contexts. It is important to note, however, that the manuscript makes no assertions regarding the validity of any simulated structures in that context.

Second, regarding the perceived low quality of figures: It appears that the reviewer has viewed only the low-resolution versions and not the high-resolution ones accessible through the hyperlinks in the manuscript. To illustrate, the relevant hyperlink to Figure 3 is repeated below, enabling the distinction between individual residues. All figures were prepared in similar high resolution.

<https://www.editorialmanager.com/pone/download.aspx?id=34439147&guid=84649e70-c626-45f3-b67e-b1ddd70ca875&scheme=1>

***Reviewer:*** *1) Major revisions

1.1) First major revision: The a-zein 20 amino-acid repeat

The cZ19C2 a-zein is a member of a 100 protein coding gene family. The a-zeins present a 20 amino acid repeated sequence which seems to play a crucial role in the protein folding and functional organization.*

**Author:** As mentioned in the manuscript (line 78 and forward) on the historical background of α-zeins, a distinctive feature of α-zein sequences is the existence of multiple degenerate ~20 AA repeats. The literature suggests that the last two-thirds of any α-zein sequence consists of such repeats, which may thus substantially influence the protein's 3D structure (lines 111). However, the definition of individual repeats remains a subject of debate, as well as the details of how they relate to secondary structure elements. As mentioned, early models averaged all putative repeats into a single consensus repeat, proposing that this repeat corresponds to a single α-helix. This study remains neutral on the validity of this approach: The modelling herein is intentionally not based on previous notions about repeat sequences and their relationship to 3D structure.

Instead, here the starting point is from a contemporary prediction method (AF2). The AF2 model demonstrates segmentation into a number of helices. Moving towards the C-terminus, each helix tends to show increased similarity to a previously published repeat sequence. This link to previous research is interesting and therefore acknowledged.

***Reviewer:*** *The author mentioned that there is no general agreement on the definition of the repeat unit, therefore the reader wishes that his work will clarify this point.*

**Author:** In line with my reply to the previous point: It is not a goal of the present study to propose alternative α-zein repeat units. Repeats from the literature are discussed in the article due to their importance in previous α-zein research and models. This study is unbiased regarding the utility of repeat units for understanding α-zein spatial structure. Hence, it remains open e.g., to the idea that published repeat units may effectively describe the structural segmentation of only a portion of the α-zein monomer, while other principles may better describe the remainder of the structure.

***Reviewer:*** *Using a multiple alignment of eight repeats, Geragthy D et al (1981) proposed a sequence consensus while Argos P. et al (1982) published an alternative consensus using a 18 repeat sequence alignment. After I consulted both referenced articles, I noticed that Geragthy’s and Argos consensi are shifted.
So, which consensus is correct ?*

**Author:** This remains unknown. As noted, the two consensus sequences are shifted. It is often hypothesized that each consensus forms a helix. If one makes this assumption, this implies glutamine repeats being positioned centrally in one case (Geraghty 1981) and terminally in the other (Argos 1982). Therefore, using the latter consensus to construct a simplified α-zein monomer model comprising several interconnected helices, the glutamine repeats act as turns, connecting adjacent consensus helices. This is the basis for several published α-zein models. In the current study, the AF2 model divides the C-terminal portion of the protein into helical segments that align more closely with (Geraghty in 1981), which is noted in the manuscript.

***Reviewer:*** *In the introduction the author quotes both Argos (line 84) and Geragthy’s (line 81) consensi. Unlike Geragthy, Argos is said to have been largely referenced (line 85). However, the author will use Geragthy’s consensus (Abstract line 31, Results line 319) for the present study without clear justification. As a result, the reader is puzzled and confused right from the start.*

**Author:** Both consensus sequences are included due to their historical significance. A number of known α-zein models are based on the structural implications of Argos's consensus sequence, in particular the positioning of glutamine residues in loops *between* helices. Conversely, there is an absence of models based on Geraghty's consensus sequence, which places glutamines *inside* helices. This reason for the relative lack of Geraghty's sequence in models remains unclear, and may be incidental.

As previously stated, this study adopts a neutral position on the validity of any consensus sequence. However, since the sequence by Geraghty *et al.* maps well onto several C-terminal helices in the AF2 model herein (one-repeat-to-one-helix) the increased focus on this sequence is justified.

To emphasize this justification, the following text has been added to the revised manuscript at Line 379: “Towards the C-terminal, this qualitative alignment highlights an increasingly direct mapping between the aligned segments and individual C-terminal helices in the AlphaFold2 model, as discussed in a later section.”

***Reviewer:*** *At the sequence level and because there is no homologous structure, a crucial task is to predict how many repeats are present in the cZ19C2 sequence, where they are localized and to which degree each of them is similar to the consensus.*

**Author:** Such extensive repeat analysis is not the focus of this article, which again remains agnostic on the importance of repeats for understanding the spatial structure of the α-zein monomer. To deliberately explore a distinct path from previous models, the AF2 model was used, which does not rely on previous assumptions about repeats or their connection to structure (Yet interestingly, the AF2 model happens to connect itself to historical repeats, given the mapping between C-terminal helices and the repeat by Geraghty et al.).

***Reviewer:*** *In figure 3a, the author proposed a representation that is too qualitative and to some extend outdated to estimate the similarity between the a-zein repeats and the consensus, he does not provide a quantitative measurement. For instance, segments 1-5 and 12 are not convincing repeat sequences because visually they poorly match the consensus.*

**Author:** Fig 3A is not intended to provide a quantitative alignment. It serves two objectives: (i) presenting the sequence of the studied α-zein and (ii) qualitatively demonstrating the increasing similarity between the helical segments in the AF2 model and the repeat proposed by Geraghty et al. towards the C-terminal.

Due to the reviewer’s comment, I realize that the qualitative nature of the alignment may not have been adequately emphasized in the initial submission. Hence, the revised manuscript includes the following modified caption for Fig 3:

“Fig 3: Consensus sequence comparison and AlphaFold2 model. A: Qualitative consensus sequence alignment. Top row (grey background) is the consensus repeat sequence by Geraghty et al. [25]. The other rows contain the mature protein sequence of cZ19C2 (UniProtKB ID: P06677) manually aligned with the consensus repeat sequence in segments of up to 20 amino acids. Green background indicates amino acids matching the consensus sequence. Black background indicates gaps. B: AlphaFold2 model. Residues corresponding to the first amino acid of each row in (A) are indicated by black labels. The α-helices I – VII are annotated in purple. Gln and Asn are shown in green and orange stick representation, respectively. The protein backbone is shown as ribbons coloured by secondary structure. α-carbons are shown as spheres coloured by sequence position (red: N-terminal, blue: C-terminal). C: pLDDT scores indicated by ribbon colour. D: Predicted aligned error (PAE) matrix.”

***Reviewer:*** *As a first major revision, the author should construct both a repeat LOGO (Crooks GE et al, 2004) (*[*https://weblogo.berkeley.edu/examples.html*](https://weblogo.berkeley.edu/examples.html)*) which is nowadays a dramatically better representation than a consensus and a a position weight matrice (PWM) (Position_weight_matrix in* [*https://en.wikipedia.org/wiki/*](https://en.wikipedia.org/wiki/)*). A quantitative similarity measurement between each cZ19C2 repeat and the PWM can be calculated using the matrix similarity score (mSS) that was described in the MATCH article (Kel A.E.et al, 2003, DOI:10.1093/nar/gkg585).*

**Author:** Again, this study remains neutral on the value of consensus repeat sequences. The mention of consensus sequences serves to (i) acknowledge their significance in the existing literature on α-zein, and (ii) highlight the correspondence with the C-terminal helical segmentation of the AF2 model in the specific case of the consensus sequence by Geraghty et al. While the methods suggested by the reviewer likely represent advancements in comparison to previous consensus approaches, it's important to clarify that this work does not pursue an exhaustive bioinformatical analysis.

***Reviewer:*** *I expect also the author to explain why he prefers Geragthy’s consensus rather than Argos one. The position of the repeats in the AlphaFold 2 (AF2) 3D model should be also clearly represented. A crucial result is to determine if AF2 predicts a-helices for the repeats.*

**Author:** I have no preference for Geraghty’s consensus sequence. Rather, this study highlights it for the single reason that it provides a clear mapping onto the helical segments in the AF2 model when moving towards toward the C-terminal. This correspondence is mentioned in the manuscript (L. 362 - 364) and is evident from Fig 3A. As mentioned in the caption for Fig 3, I have labeled (using black text) the positions in the 3D structure Fig 3B corresponding to the first amino acid of each row in the qualitative alignment to Geraghty’s consensus in Fig 3A. This demonstrates that rows in this alignment initiated by NPA, NRA, or DPA maps on to the start of a new helix in the AF2 model. Since Geraghty's consensus starts with NPA, this qualitative alignment establishes an interesting connection to previous literature. Highlighting this connection is a key objective of the figure.

*1.2) Second major revision: quality of the a-zein AF2 predicted 3D model*

***Reviewer:*** *At the structural level, the author starts the conformation investigations using a 3D molecular model of the cZ19C2 protein generated by ColabFold, i.e. a web interfaced AF2 server and hypothesizes that deep learning might compensate for the lack of a-zein homologous protein experimental structures in the Protein Data Bank (PDB). I am afraid the author assumed too much.*

**Author:** The actual wording I used was more restrained than suggested by the reviewer, and served only to describe one of several considerations that shaped my evaluation of the AF2 model as more suitable than the alternatives (homology or ab initio models). I repeat the original lines 203-204 in the manuscript below, with cautious terms underlined for emphasis for the sake of this reply:

“It was hypothesized that this level of data integration might partially compensate for the lack of homologous experimental structure templates for α-zein.”

Nevertheless, this cautious expression of optimism for the AF2 model has no strong bearing on the study: *Any* predicted α-zein model remains fundamentally speculative, given the impossibility of validation against even moderately homologous experimental high-resolution structures. In other words, regardless of starting point, a significant leap of faith is always needed to initiate molecular modelling studies on α-zein. This study does not ignore or conceal this inherent uncertainty. However, as outlined in the manuscript and reiterated in the review points below, specific additional features (or “heuristics”) of the AF2 model suggest it may be more suitable for the conformational studies of the solution states discussed here, when compared to the typically more compact models from other methods.

***Reviewer:*** *Since AF2 breakthrough at CASP14, it has been known that AF2 generates good, bad and ugly models (see J.M. Thornton et al, 2021, DOI:10.1038/s41591-021-01533-0). The cZ19C2 a-zein model belongs to the group of bad or ugly protein 3D structure predictions****.***

**Author:** First, the fact that the cZ19C2 α-zein model is uncertain is a premise accepted by this study, as must be the case in any α-zein study due to the lack of high-resolution experimental structures for reference. Second, since any predictive approach yields uncertain models of this atypical protein, the decision to use the AF2 model of α-zein for further studies was supported by curated features or heuristics (detailed below) rather than by confidence metrics (e.g., ProSA scores, AF2 pLDDT and PAE and their derived “good”, “bad”, “ugly” classifications.). The rationale for this was: I posit that the meaning and relevance of protein confidence metrics is ambiguous when the protein is very atypical (α-zein is) and/or when the environment in which the protein is studied deviates substantially from the biological environment (the case here). As an example, the models generated with the *ab initio* method C-QUARK (Fig D in S2 Appendix) are relatively compact models, which are ostensibly “better” relative to the AF2 model if judged only by the ProSA z-score. But better in what environment? Their compactness suggest they could perhaps be examined for representing the solid-state α-zein conformations inside protein bodies. Yet, they appear less likely candidates for solution states, since previous SAXS and NMR studies do not indicate compact forms. Hence, despite their apparently better metrics, such compact models were not chosen for MD simulations in this study. In contrast, the AF2 model, despite its low pLDDT and PAE metrics, has several characteristics that, in my assessment, indicate its suitability for the current study. The main features are summarized here in short form, with references to the actual lines in the manuscript:

(i) Wider (deep learning based) data integration in contrast to earlier α-zein models (Manuscript lines 202 – 205).

(ii) Overall loose protein packing, qualitatively consistent with the absence of compact conformations in previous SAXS and NMR solution zein studies (Manuscript lines 332 – 334).

(iii) Correspondence between the C-terminal helical division and the consensus sequence by Geraghty *et al.* (Manuscript lines 362 – 364).

Lastly, it's worth noting that it has been a suggested [GUO_2022] that the AF2 pLDDT and PAE metrics, aside from their conventional use as confidence metrics, might be construed as indicators of residue flexibility similar to how root mean squared atomic fluctuations (RMSF) are interpreted. It cannot be definitively asserted, of course, whether this is an accurate interpretation of these metrics in the current study. Nonetheless, given the substantial literature evidence on the flexibility of α-zein, this possibility could be explored in future research.

[GUO_2022] Guo H-B, Perminov A, Bekele S, Kedziora G, Farajollahi S, Varaljay V, et al. AlphaFold2 models indicate that protein sequence determines both structure and dynamics. Scientific Reports. 2022;12(1):10696. doi: 10.1038/s41598-022-14382-9.

**Reviewer:** *I think it is reckless to embark on MD simulations and conformation studies using a 3D model that shows such a poor quality unless the author proved that the MD were long enough to simulate the 3D folding of the a-zein polypeptide primary sequence. Since the author did not claim it, I understand it was not his ambition.*

**Author:** The focus was not to study conformations of a model representing a putative native-like state of the α-zein (i.e., in its solid-state environment inside protein bodies). Instead, the aim is to contribute to the study of conformational ensembles across different relevant (but non-native) solution models, as stated in the manuscript (lines 194 to 196), reiterated here:

“This implies that a single native structure, if such exists, is unlikely to represent the diverse array of α-zein conformations in practical applications. Therefore, the main goal of the present study is not to suggest a single native-like state of zein, but rather to contribute to charting the conformational landscape of α-zein in various solution environments.”

In light of this stated goal, I assert that it is not reckless to analyze the behavior and conformations of the loosely packed AF2 starting point in solutions. In extension of my comments on the reviewer’s previous point, I would argue the opposite: Solely relying on protein quality metrics to assess the adequacy of a model for a protein as atypical as α-zein would indeed appear reckless.

***Reviewer:*** *Two AF2 metrics warned that the model was bad, first the per-residue LDDT scores and second the predicted aligned error matrix (PAE) matrix, the latter was totally absent in the manuscript. To study the conformation landscape of a protein, it is crucial that* *a reliable model or experimental structure is available. For the a-zein it is definitely not the case.*

**Author:** I have argued against the reviewers' insistence on the primacy of AF2 metrics in a previous point.

The reviewer adopts an unusually restrictive position by insisting on the necessity of a reliable model or experimental structure as a fundamental prerequisite for MD conformational studies. Such a perspective is not universally accepted or applied. For example, intrinsically disordered proteins (IDPs) are widely studied using MD simulations, despite lacking “reliable” starting points, see e.g. [Wang_2021] or [Shrestha_2021]. While α-zeins are typically not considered IDPs, they share the circumstance of lacking experimental high-resolution structures. Consequently, their study must also rely on a speculative starting point.

In my opinion, this lack of certainty should not invalidate careful exploratory research that clearly states its limitations while aiming to contribute to the incremental elucidation of possible conformations of an atypical and important protein.

[Wang_2021] Wang W. Recent advances in atomic molecular dynamics simulation of intrinsically disordered proteins. Physical Chemistry Chemical Physics. 2021;23(2):777-84. doi: 10.1039/D0CP05818A.

[Shrestha_2021] Shrestha UR, Smith JC, Petridis L. Full structural ensembles of intrinsically disordered proteins from unbiased molecular dynamics simulations. Communications Biology. 2021;4(1):243. doi: 10.1038/s42003-021-01759-1.

***Reviewer:*** *The per-residue LDDT scores are low or very low. Only the C-terminus three helix bundle may be valuable – see below-. First, I request the author to create a main figure that will present a cartoon of the 3D model with each residue colored according to its LDDT score. The reader must know if the AF2 model can be trusted and this information must appear graphically on a structural figure in the main manuscript. Second, the author must also show in the same figure the PAE that is generated by AF2 to determine how reliable are inter-helix distances in the model.*

**Author:** In response to the reviewer's request, I have incorporated both the pLDDT-color coded backbone of the AF2 model and the corresponding PAE matrix in the lower panel of the revised Figure 3 as subfigures Fig 3C and Fig 3D. I view this as a reasonable addition to the figure to enhance transparency, despite my disagreement with the reviewer's insistence on the importance of these metrics in the specific context of this study. Therefore, beyond incorporating the new subfigures, I have refrained from including further discussion of these metrics in the revision.

***Reviewer:*** *Using the PAE of the a-zein AF2 model (see P06677 record in the EBI AlpaFold database), the matrix is largely white, i.e. AF2 does not know how to organize spatially the secondary structures. It is unfortunate that the author did not take into account the PAE as a second bad quality warning of the model. It can be observed that the matrix is slightly greener in the 160-225 residue square, i.e. the fold of the C-terminus three helix bundle. This region is slightly more reliable than any other part of the model which is totally unusable for further analysis. As a consequence, the MD simulations and the conformation study should be carried out only on the helix bundle. This is my major remark since I consider that outside the bundle the quality of the AF2 model is so bad that running MD simulations makes no sense.*

**Author:** The arguments I have presented in response to the points above reflect my strong opposition the reviewer’s suggestion of redoing the entire study on a truncated AF2 model. The simulation trajectories for the full AF2 model, encompassing both ordered and less ordered regions, stand as the primary outcome of this study. However, I am open to the idea that further examination of the C-terminal region, particularly including the more compact three-helix bundle, could be interesting. This aligns with the feedback from another reviewer.

Since the C-terminal helical bundle superficially resembles a coiled-coil, MD studies were done to analyze its structure in an aqueous environment as typical for coiled-coils. To this end, I have carried out an additional 1 µs MD simulation in TIP3P water on the C-terminal residues (N144-F219) and included the analysis of the results with the revision in the new S6 Appendix. In brief, the three-helix bundle becomes more compact, but is largely preserved throughout the MD simulations, demonstrating substantial retention of the initial α-helical configuration assigned by AF2.

***Reviewer:*** *1.3) Third major revision: Write a discussion

The article shows no discussion paragraph. The author is expected to discuss his results, for instance the quality of the AF2 model.*

**Author:** The manuscript already contains a combined 'Results and Discussion’ section (Line 306), covering all aspects deemed relevant. Specifically, the AlphaFold2 model is discussed (L. 331) and the low lDDT scores are mentioned (L. 335).

***Reviewer:*** *1.4) Fourth major revision: number of figures in the main manuscript and SI

For clarity and concision, reduce to 7 or 8 the number of figures in the main manuscript while 15 is recommended not to be exceeded in SI.*

**Author:** I restate my response to another reviewer's query concerning the number of figures: I acknowledge the reviewer’s opinion about reducing the number of figures, but I would like to note that my use of figures aligns with the journal's liberal figure policy i.e.:

“There are no restrictions on word count, number of figures, or amount of supporting information.” (https://journals.plos.org/plosone/s/submission-guidelines).

The number of figures can only be reduced by (a) excluding figures or (b) by merging multiple figures. I have already considered both approaches while preparing the manuscript. For instance, the reviewer suggests merging Figures 4 – 8, which already consist of subfigures. Further merging of Figures 4 – 8 would lead to a very large figure, even after excluding certain elements that could be considered redundant. The resolution of such a figure would be lower relative to the current individual figures, since the journals “Figure File Requirements” states a maximum figure file size of 10 MB. In my opinion, sacrificing figure resolution for space is not a satisfactory tradeoff here. Similarly, omitting figures solely to condense the manuscript seems to contradict the relatively free-format ethos of the journal. Given these considerations, I have decided not to reduce the number of figures.

***Reviewer:*** *2) Minor revisions

2.1) Abstract:

In the cell, what is the natural environment of a-zein, i.e. cytoplasm, membrane, extracellular ?

What is glycidyl methacrylate ? Do not assume that readers will know what is this compound for.*

**Author:** The revised abstract has been reformulated in consideration of feedback from both this reviewer and another. It now incorporates information on the role of glycidyl methacrylate and provides details about the natural environment of α-zein. To further emphasize the role of glycidyl methacrylate as a crosslinking reagent, I have changed the original heading of the section “Interactions with Glycidyl Methacrylate” to “Interactions with a Crosslinking Reagent” in the revision.

***Reviewer:*** *2.2) Expressed sequence tags (ESTs)

Unlike RNA-seq reads, ESTs are not correlated with gene expression levels, they enable the biologist to determine which genes are expressed in a tissue sample and only provide a qualitative information on which genes are highly expressed. Therefore “The top-three expressed random selected clones measured by percentage expressed sequence tags (ESTs)” sentence is not rigorous and an alternative formulation should be found such as
“Using EST, az19B1, az19B3, and az22z1 were found among abundantly expressed genes”.*

**Author:** The data is taken from the publication [The Plant Cell, Volume 13, Issue 10, October 2001, Pages 2297–2317, <https://doi.org/10.1105/tpc.010240>] and I essentially use their nomenclature and interpretation. In Figure 1 of that article, “percent expressed sequence tags” shows the “transcript levels” of various zeins (including the α-zeins mentioned in this manuscript) reflected by their percentage among randomly chosen clones from 15- to 40-DAP endosperm specific cDNA libraries. In that figure, the transcript levels follow the ranking az19b1 >> az19b3 > az22z1. To the best of my knowledge these data and interpretations have not been challenged. I therefore see no rationale for changing the wording.

***Reviewer:*** *2.3) Figure 1

The percentage values that are indicated on Figure 1 tree should be withdrawn since the percentages are not correlated with gene expression levels. Mark the three selected a-zein with a symbol to indicate that they are among abundantly expressed genes in B73 endosperm, it would be enough.*

**Author:** As these percentages have been published, and to my knowledge not challenged, I see no reason for removing them from the figure. See my answer to the previous point.

***Reviewer:*** *In the tree, why do the cZ19C2 leaf shows two UniprotKB identifiers ? Is it a sequence redundance in UniprotKB ? Please select only one for clarity.*

**Author:** Yes, this redundant sequence identifier was included for completeness. However, I agree with the reviewer's suggestion to omit the ID from the tree for clarity, and the revised version has been modified accordingly.

***Reviewer:*** *Was the a-zein tree built using a protein sequence alignment (which program ?) and a phylogenetic algorithm (NJ, Bayes, ML, MP) ? In the legend, please give more information on the method. The tree lacks a scale to understand what are the numerical values on the branches.*

**Author:** The reviewer is thanked for highlighting the missing information about the tree. The revised caption for Fig 1 is given below:

“Fig 1. Topological tree for α-zein protein sequences. Zein names are from the studies [23, 29, 30]. UniProtKB IDs are given in brackets. Expressed sequence tag (EST) percentages are given for the three most expressed randomly chosen clones in B73 endosperm [30]. The cZ19C2 zein studied here is indicated by a grey background. The tree excludes signal peptides. The tree was built with MEGA11 [32] using a multiple sequence alignment of the mature α-zein sequences carried out with the integrated CLUSTAL W [33]. The Maximum Likelihood method and the JTT matrix-based model were used to infer the evolutionary history [34]. The tree with the highest log likelihood (-2686.46) is shown. The initial tree(s) for the heuristic search were automatically obtained using the Neighbor-Join and BioNJ algorithms on a pairwise distance matrix estimated with the JTT model. The topology with the highest log likelihood value was selected. This analysis included 17 amino acid sequences, with a total of 276 positions in the final dataset. Branch lines do not scale with branch lengths; however, the branch lengths, measured in the number of substitutions per site, are displayed next to the branches.”

***Reviewer:*** *2.4) Figure 2:

What do numbers in brackets represent ? Please write it in the figure legend.*

**Author:** This is already stated in the caption (Line 106 in the original submission).

***Reviewer:*** *2.5) Figure 3A:

The residue offsets do not correspond to sequence Uniprot-ID:P06677.
I understand the author trimmed off the Nter peptide signal and renumbered the residues starting from Ala31. This is confusing when the readers wish to localize precisely the repeats in the Uniprot-ID:P06677 sequence. Renumber the residues in figure 3A using P06677 full-length protein sequence.*

**Author:** I acknowledge that from a purely bioinformatical perspective there could be a motivation to renumber the residues in Figure 3A using the full-length protein sequence of Uniprot-ID:P06677 including the signal peptide. On the other hand, signal peptides are normally ignored when numbering residues in structures of proteins and peptides. The decision to omit the N-terminal peptide signal and renumber the residues from Ala31 was made in accordance with this widely used practice to focus on the structure of the mature protein (and not the preprotein).

***Reviewer:*** *2.6) Figure 4 to 8:

The structural figure resolution is too low to clearly distinguish the conformations.

The helices are colored in magenta but some regions are colored in blue or yellow. The author does not explain in the legend what does the blue and yellow colors mean.

Figure 7E, 7H, 8B, 8E and 8H are particularly messy.*

**Author:** The reviewer must have viewed the low-resolution versions of the figures rather than the downloadable full versions hyperlinked in the manuscript. The full resolution figures reveal sufficient detail for their intended purpose. Specifically for Figures 4 – 8, the intention is to visualize qualitative trends of the MD *ensembles*, showing their relative (dis)order, rather than enabling the distinction between individual conformers. Therefore, the relative "messiness" in Figures 7E, 7H, 8B, 8E, and 8H, which can be more accurately described as conformational heterogeneity, is precisely the insight these figures aim to convey. Other figures (e.g. Fig 14) serve the purpose of visualizing specifics of individual structural MD snapshots.

However, I appreciate the feedback regarding the lack of an explanation of the ribbon color coding. The coloring is according to secondary structure assigned by the STRIDE program as implemented in VMD. I have therefore included the required legend (which applies to Fig 3 – 8, 12 - 15) in Fig 3 of the revised manuscript.

***Reviewer:*** *3) Reviewing conclusion:

Because of the tremendous amount of work which has been produced by the author I would feel guilty if I suggested the manuscript to be rejected. However, there are serious concerns about the reliability of the AF2 model which was used as the starting structure for MD simulations and major revisions are required.*

**Author:** Hopefully my responses to points above have made it clear that the premise of using the AF2 model in the present context is justified.

***Reviewer:*** *On one hand, claiming that the a-zein shows a packed conformation in water sounds to me as an expected consequence of the poor quality of the AF2 model. Indeed, the three MD seeds of figure 4 in 100% water ends with protein conformations that cannot be superposed even locally, i.e. helices are messily packed. Don’t we expect some kind of structural ordered organization of the protein to understand biopolymer properties ?*

**Author:** While often assuming well-organized structures, it is also recognized that proteins display dynamic behavior, see [Lane_2023], and can exist in various structural states. The degree of structural heterogeneity can vary considerably, with intrinsically disordered proteins, as mentioned earlier, serving as notable examples with little permanent organization. It is an open question whether α-zein natively adopts a single structural minimum or shares similarities with intrinsically disordered proteins. However, the present study specifically concentrates on α-zein in non-native solution environments, reporting on potential structural ensembles. The distinct compact conformational states observed in water simulations for each seed are not proposed as definitive models of actual states but are presented as possible ensembles for further investigation. To this end, PDB coordinates for these ensembles are available for download via the link to Kaggle provided with the original manuscript.

[Lane_2023] Lane TJ. Protein structure prediction has reached the single-structure frontier. Nature Methods. 2023. doi: 10.1038/s41592-022-01760-4.

***Reviewer:*** *On the other hand, to claim that the polypeptide elongates in ethanol reminds me the protein denaturing effect of the solvent. Is it an original result ? I would say no.*

**Author:** I don't claim originality regarding the denaturing effect of ethanolic solvents on proteins in general. Instead, this study reports on this specific α-zein's expansion observed in MD simulations in ethanolic solvent models, aligning with certain experimental findings. The study also offers insights into the effects of varying ethanolic concentrations.

*Therefore major revisions must be carried out and I invite the author to rethink the article by focusing the analysis on the C-terminus three a-helix bundle only.*

**Author:** In summary, the primary outcome of this study is represented by the simulation trajectories of the full AF2 model, incorporating both structured and less organized regions. These trajectories represent *possible* ensembles for the investigated α-zein in simulated ethanolic environments, which, while non-native, approximate technologically relevant solution systems for α-zeins.

Furthermore, a new appendix has been incorporated into the revision, presenting an additional 1 μs MD simulation and analysis focused on the protein's C-terminal region, including the three-helix bundle. This illustrates the dynamic conservation of this region, even when isolated from the rest of the protein. These findings may inspire further studies, potentially with practical applications in mind for the three-helix bundle (e.g. as a coiled-coil mimic).

***Reviewer #4****: The authors have performed an interesting work on Conformations of a highly expressed Z19 α-zein studied with AlphaFold2
2 and MD simulations. However, a few queries need to be addressed.*

**Author:** I thank the reviewer for the feedback on the manuscript.

***Reviewer:*** *1. The title seems to be less scientific. The authors can reframe the title as "Insights into Z19 α-Zein's Structural Dynamics: AlphaFold2 and MD Simulation Approaches".*

**Author:** It is not clear to me what the reviewer means by "less scientific" in this context. Judging from the reviewer's suggestion for the revised title, I speculate that the reviewer’s concern may relate to the use of "highly expressed" in the title. However, I believe there is convincing published evidence for relatively high expression of the α-zein studied (see Woo et al., Plant Cell. 2001 Oct; 13(10): 2297–2318). Hence, I choose not to alter the title, as I am uncertain of the purpose it would serve.

***Reviewer:*** *2****.*** *What motivated the selection of ethanol concentrations (0, 3, 23, 50, and 100 mol%) in the MD simulations? Were these concentrations based on previous studies or theoretical considerations?*

**Author:** The reviewer correctly points out the need for clarification regarding the selection of ethanol concentrations. In response, I have incorporated the following clarification in the revised manuscript:

“The set of 0, 2, 23, 50, and 100 mol% ethanol concentrations in water were selected to encompass simulations with ethanol percentages typical of experimental solution systems, i.e., 60 – 95 vol% ethanol [50] as well as concentrations approaching the solubility limits of α-zein (i.e., approximating pure water or pure ethanol). The latter concentrations, representing extreme conditions, were included with the expectation that they might perturb the protein structure, thus extending the types of conformations sampled”

Due to the reviewer’s comment, I discovered that a concentration of 3 mol% was wrongly stated in the abstract and in the conclusion. This has been corrected, now stating the actual concentrations used, i.e., “0, 2, 23, 50, and 100 mol%”

***Reviewer:*** *3. What is the significance of the proposed work? What is the cause behind employing various force fields in MD simulation?*

**Author:** The final paragraph of the conclusion outlines the key significance of this research. To address the reviewer's query, I rephrase the essence of that paragraph here, adding more detail: The research contributes to the study of the conformational behavior of the technologically significant maize protein α-zein in ethanolic solvent systems commonly used in experimental contexts. Due to the absence of high-resolution experimental α-zein structures, numerous disparate molecular models have been proposed over the years to represent the structure of these proteins. By combining for the first time AlphaFold2 and extensive MD simulations to study an α-zein (cZ19C2), this work aims to further explore the landscape of possible α-zein conformations. Detailed knowledge of α-zein’s different conformations in relevant environments may ultimately inform new strategies for chemical or enzymatic modification of these proteins, possibly enabling their broader applications in biomaterials, biomedicine and biotechnology. The study briefly ventures into this direction by examining the interaction between the common crosslinking reagent, glycidyl methacrylate (GMA), and α-zein in water. In water, a compact conformation of α-zein formed favorable interactions with GMA, aligning qualitatively with literature findings showing hydrophilic enhancement of reactions between GMA and α-zein.

Regarding the choice of force fields: For GROMACS simulations, The OPLS-AA/L force field was chosen for its long history and broad applicability in biomolecular simulations and it served as the primary all-atom force field in this study. However, given the exploratory nature of the research, which involves the loosely packed AlphaFold2 starting point, reference simulations were conducted with the newer force field ff99SB*-ILDN at 100 mol% water and 24 mol% ethanol concentrations. The reference simulations served as a supplementary evaluation of some trends in the OPLS-AA/L simulation series. One aspect examined was whether the ff99SB*-ILDN force field indicated a different propensity for β-sheet formation in the α-zein model. This is relevant in light of the conflicting findings in the literature regarding β-sheet content in α-zein. The ff99SB*-ILDN simulations concurred with the low β-sheet content in the OPLS-AA/L simulations at comparable concentrations. For Desmond simulations, the default OPLS3 force field was used. Desmond simulations were used chiefly for simulations on (a) the six-copy α-zein system and (b) the examination of α-zein interactions with the crosslinking agent glycidyl methacrylate (GMA) in water. The OPLS3 force field was considered appropriate in both contexts, given its parametrization for both small molecules and proteins. Finally, for coarse-grained (CG) simulations in GROMACS, the MARTINI 2.2 force field was chosen due to its popularity in large-scale CG biomolecular simulations.

***Reviewer:*** *4. How was the reliability or accuracy of the AlphaFold2 model assessed prior to commencing the molecular dynamics simulations?*

**Author:** As mentioned in the manuscript (lines 193 to 196), the primary objective of this study was not to model a “native-like state” of α-zein representative of its physiological solid-state environment in protein bodies. Several previous α-zein models have already targeted the understanding of this state. This study deliberately deviates from this path, by concentrating on conformational ensembles in relevant but non-native solution environments.

In light of this objective (and as explained in my response to another reviewer), I argue that evaluating the appropriateness/reliability of the initial structural model used in MD simulations here mainly through protein model reliability/quality metrics like pLDDT, PAE, and similar measures has limited utility and may even be counterproductive (for example, by favoring compact structures that do not represent the studied conditions). This assertion arises from the atypical nature of both the protein (α-zein) and the studied environment (primarily ethanolic solvent). Instead of relying on such metrics, the selection of the AlphaFold2 model was supported by several attributes I considered important for the specific context under study. They are outlined here:

(i) Wider (deep learning based) data integration in contrast to earlier α-zein models (Manuscript lines 202 – 205).

(ii) Overall loose protein packing, qualitatively consistent with the absence of compact conformations in previous SAXS and NMR solution zein studies (Manuscript lines 332 – 334).

(iii) Correspondence between the C-terminal helical division and the consensus sequence by Geraghty *et al.* (Manuscript lines 362 – 364).

Nevertheless, to enhance transparency, the AlphaFold2 metrics (pLDDT scores) were displayed in S1 Fig and discussed in the main text (Lines 335-336). Additionally, in response to another reviewer's suggestion to integrate pLDDT scores and the predicted aligned error (PAE) matrix into the main text, both have been incorporated into the revised Fig 3 as subfigures C and D.

***Reviewer:*** *5. Could you provide insights into why extended conformations prevailed at higher ethanol concentrations and the impact of these conformations on the protein's structure and function?*

**Author:** While a detailed characterization of the molecular interactions between ethanol and α-zein holds relevance and the potential for interesting insights, it was deemed to be beyond the scope of this study. However, the observed extended α-zein conformations in both literature (e.g., in SAXS/NMR experiments in water/alcoholic solutions) and the simulations in this study are likely caused at least partially by the substitution of stabilizing intra-protein sidechain-sidechain interactions with sidechain-ethanol interactions. Such interactions are commonly suggested to contribute to the denaturing effect of alcohol on proteins in general and were observed in the simulations herein (results not shown). Future extensions of this study may analyze the spatiotemporal distribution of such interactions as a function of ethanol concentration and their correlation with α-zein extension. Additionally, such studies may aim to clarify the extent to which the observed extension α-zein aligns with the denaturation route for more typical proteins in ethanolic solvent.

The only known biological function of α-zein is to act as nitrogen storage for germinating corn seeds. The conformation of α-zein likely influences the molecular packing within protein bodies in the corn endosperm. Published models for α-zein within protein bodies, including the consensus models discussed in the manuscript, propose that compact α-zein monomer conformations lead to denser monomer-monomer packing. Conversely, extended conformations may be expected to result in less dense packing. The specific impact of packing variations on the biological function of the protein remains a subject of speculation. However, from a protein engineering perspective, the extended and compact conformations of α-zein are expected to impact chemical/enzymatic access to residues and may hence influence protein engineering strategies.

***Reviewer:*** *6. Beyond biomaterials, are there other industrial or environmental applications where this detailed map of α-zein conformational variation could be particularly relevant or impactful?*

**Author:** Zeins, including α-zeins, have a rich history of applications across various fields, as detailed in the literature and briefly outlined in the manuscript's introduction. Nevertheless, as for other proteins, rational modification of α-zein requires detailed structural elucidation. This includes conformational characterization under relevant conditions in order to guide structural modifications for new applications. Therefore, it is possible that this study, combined with existing and future investigations, may play a role in advancing the use of α-zein for biomaterials as well as in other fields, such as therapeutics, food ingredients, and beyond.

The simulations herein draw attention to certain aspects that may warrant future examination for new applications. In particular, one may speculate that the C-terminal α-helical bundle predicted by AlphaFold2 and found relatively stable in the MD simulations, might present an interesting alternative to scaffolds based on coiled coil motifs. Structural designs incorporating the α-helical bundle may perhaps fulfil similar roles as designs based on coiled coils (e.g., biosensors, heavy-metal binders) but with another biocompatibility profile (zein is FDA approved for use in biomedicine). Similarly, regions found to be more dynamic in the simulations herein may be investigated for application where such dynamics can be actively leveraged, perhaps in stimulus responsive peptide systems [Chockalingam] for biosensors or drug delivery.

[Chockalingam] Chockalingam K, Blenner M, Banta S. Design and application of stimulus-responsive peptide systems. Protein Engineering, Design and Selection. 2007;20(4):155-61. doi: 10.1093/protein/gzm008.

***Reviewer:*** *7. Why did the authors focus on backbone RMSD? What is the reason behind employing 400 ns simulation period?*

**Author:** Backbone RMSD is a typical measure of structural changes in MD simulations. Herein RMSD was found to be useful in conjunction with SASA and radius of gyration for identifying and discussing α-zein conformational variation.

The default simulation period of 400 ns was chosen to enable exploration of significant conformational changes of the loosely packed AlphaFold2 starting point. This choice aimed to strike a reasonable balance between computational resources and the extent of conformational sampling. Of course, deciding on a specific simulation duration is subjective since, in practice, there is no upper limit for what may be considered adequate. This study acknowledges the potential value of longer simulations, and hence, as described in the manuscript, selected simulations were extended substantially beyond their initial 400 ns period.

***Reviewer:*** *8. Explain how did the authors declare collapse and structural expansion based on MD simulation*

**Author:** I believe this is explained in the manuscript: From line 430 onwards, the term "collapse" is used to describe the fast rearrangement of the initial AlphaFold2 structure towards more compact globular forms in simulations with high water concentration. In contrast, the term "expansion" denotes the structural expansion (relative to water) in simulations with high ethanol content, as outlined from Line 438 onward. As discussed, "collapse" and "expansion" are evident from the inspection of MD structural snapshots and also from the relative decreases and increases, respectively, in the timeseries for both SASA and radius of gyration.

[NOTE: If reviewer comments were submitted as an attachment file, they will be attached to this email and accessible via the submission site. Please log into your account, locate the manuscript record, and check for the action link "View Attachments". If this link does not appear, there are no attachment files.]

While revising your submission, please upload your figure files to the Preflight Analysis and Conversion Engine (PACE) digital diagnostic tool, [https://pacev2.apexcovantage.com/](https://eur02.safelinks.protection.outlook.com/?url=https%3A%2F%2Fpacev2.apexcovantage.com%2F&data=05%7C02%7Cnjc%40chem.ku.dk%7Cc02134b5f56b41ee7aa608dc0be62eea%7Ca3927f91cda14696af898c9f1ceffa91%7C0%7C0%7C638398330752024687%7CUnknown%7CTWFpbGZsb3d8eyJWIjoiMC4wLjAwMDAiLCJQIjoiV2luMzIiLCJBTiI6Ik1haWwiLCJXVCI6Mn0%3D%7C3000%7C%7C%7C&sdata=799Rzzkzy4kg908TE90KAVyeGahMvdm3Whj%2BRsbehYw%3D&reserved=0). PACE helps ensure that figures meet PLOS requirements. To use PACE, you must first register as a user. Registration is free. Then, login and navigate to the UPLOAD tab, where you will find detailed instructions on how to use the tool. If you encounter any issues or have any questions when using PACE, please email PLOS at [figures@plos.org](mailto:figures@plos.org). Please note that Supporting Information files do not need this step.

*In compliance with data protection regulations, you may request that we remove your personal registration details at any time.* [*(Remove my information/details)*](https://eur02.safelinks.protection.outlook.com/?url=https%3A%2F%2Fwww.editorialmanager.com%2Fpone%2Flogin.asp%3Fa%3Dr&data=05%7C02%7Cnjc%40chem.ku.dk%7Cc02134b5f56b41ee7aa608dc0be62eea%7Ca3927f91cda14696af898c9f1ceffa91%7C0%7C0%7C638398330752024687%7CUnknown%7CTWFpbGZsb3d8eyJWIjoiMC4wLjAwMDAiLCJQIjoiV2luMzIiLCJBTiI6Ik1haWwiLCJXVCI6Mn0%3D%7C3000%7C%7C%7C&sdata=zlRsDuDlG7%2FFWI8yhx1xysC882%2FkODI4uWV17zl3Hvs%3D&reserved=0)*. Please contact the publication office if you have any questions.*
